# Hybrid Nanofiber-Based Atmospheric Water Harvesters: Sunlight-Driven Operation in Low-Humidity and Low-Illumination Environments

**DOI:** 10.1021/acsnano.5c03322

**Published:** 2025-05-27

**Authors:** Yi Hu, Yu Chen, Jianing Xu, Wanli Cheng, Yi Lu, Guangping Han, Orlando J. Rojas

**Affiliations:** 1 Key Laboratory of Bio-Based Material Science and Technology (Ministry of Education), 47820 Northeast Forestry University, Harbin 150040, P.R. China; 2 Bioproducts Institute, 8166 University of British Columbia, 2385 East Mall, Vancouver, BC V6T 1Z4, Canada; 3 Department of Chemical and Biological Engineering, 8166 University of British Columbia, 2360 East Mall, Vancouver, BC V6T 1Z3, Canada; 4 Institute of Process Engineering, Chinese Academy of Sciences, Beijing 100190, PR China; 5 Department of Wood Science, The University of British Columbia, 2424 Main Mall #2900, Vancouver, BC V6T 1Z4, Canada; 6 Department of Chemistry, The University of British Columbia, 2036 Main Mall, Vancouver, BC V6T 1Z1, Canada; 7 Department of Bioproducts and Biosystems, Aalto University, Vuorimiehentie 1, P.O. Box 16300, 02150 Espoo, Finland

**Keywords:** cellulose nanofiber, ambient drying, aerogel, atmospheric water harvesting, hygroscopic materials

## Abstract

Aerogels incorporating hygroscopic salts have been widely explored for atmospheric water harvesting (AWH). However, the scalability of these sorbents remains limited due to their reliance on energy-intensive and time-consuming drying methods such as lyophilization or supercritical drying. Here, we present a simple and scalable approach to drying hydrogels with desirable AWH properties using a freezing process followed by solvent exchange and thawing at room temperature. Our system consists of cellulose and silica nanofibers, forming hybrid xerogels with ultralow density (10.86 ± 0.32 mg cm^–3^), high specific surface area (104.22 m^2^ g^–1^), excellent water stability, and mechanical strength. By incorporating carbon-based photothermal materials and lithium chloride as a hygroscopic salt, the xerogels achieve exceptional water uptake capacities ranging from 0.90 to 3.21 g g^–1^ across a relative humidity (RH) range from 15 to 75%. Under natural sunlight, the AWH xerogel produces water at a rate of 1.17 g g^–1^ day^–1^. These results highlight a sustainable and scalable AWH strategy, leveraging ambient-dried xerogels as an energy-efficient solution to mitigate water scarcity.

## Graphical abstract

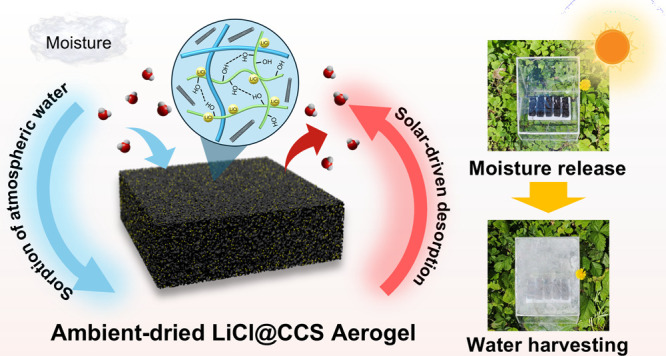

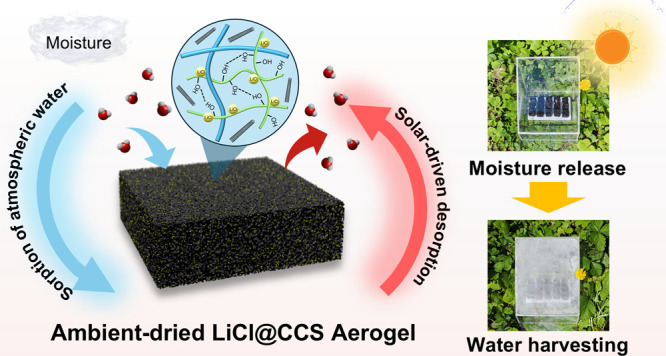

## Introduction

Water scarcity is a pressing global challenge, directly impacting over 2 billion people and disrupting migration patterns and food security.^1,2^ The United Nations estimates that by 2025, 1.8 billion people will live in regions experiencing absolute water scarcity, while two-thirds of the global population will face water stress.^3^ In many developing regions, existing water treatment technologies remain impractical due to high infrastructure requirements and energy demands.^4,5^ As a result, atmospheric water—an underutilized freshwater resource—has emerged as a sustainable alternative. Notably, the atmosphere contains approximately 13 trillion m^3^ of water, equivalent to the annual flow of the Amazon River.^6–8^ This makes atmospheric water harvesting (AWH) an attractive solution due to its environmental adaptability and minimal dependence on geological constraints.^9,10^

Hygroscopic materials, such as deliquescent salts (*e.g*., lithium chloride,^11^ calcium chloride,^12^ and magnesium chloride^13^) and metal–organic frameworks (MOFs),^14^ have demonstrated high moisture absorption capacity.^15^ For instance, lithium chloride can absorb up to ∼1.9 g g^–1^ of water vapor at 30% relative humidity (RH).^16^ However, the hygroscopic nature of LiCl and similar salts leads to particle aggregation, forming passivation layers that reduce sorption rates, efficiency, and cycling performance.^17^ To overcome these limitations, salts and MOFs are often incorporated into highly porous supporting matrices,^18^ such as cryogels and aerogels. Unfortunately, these materials require energy- and time-intensive processing, consuming over 10 kW h per kilogram of material during freeze-drying alone.^19,20^ Additional procedures, such as repeated freeze-drying for hygroscopic material loading, further increase costs and complexity.^21^

Ambient pressure drying^22^ reduces structural damage of porous scaffolds when using low-surface-tension solvents. However, simplifying processing while balancing the low density and mechanical strength remains challenging. Scaffold reinforcement can be achieved through ionic,^23,24^ covalent,^25,26^ or physical cross-linking,^27,28^ while capillary stress can be mitigated by adding surfactants^10^ or replacing water with low-surface-tension fluids such as ethanol,^29^ isopropanol,^30,31^ or acetone.^32^ However, the multistep processing, energy demands, and waste residues involved in typical AWH systems remain prohibitive.

In this study, we introduce a freezing-ethanol solvent exchange-thawing and ambient drying (FESTA) approach for synthesizing low-density xerogels without the need for freeze-drying or supercritical drying. By replacing the liquid phase of the gel with air, we achieve minimal structural collapse, preserving the nanoscale porous network and yielding an ultralow-density material with high surface area. In light of these properties and the widespread usage of the term, we hereafter refer to these materials as ″aerogels″. To demonstrate their potential for AWH, we incorporated hygroscopic lithium chloride and carbon nanotubes into the aerogel matrix (LiCl@CCS), enabling water sorption and photothermal conversion while maintaining structural stability during solar-driven AWH operations.

## Results and Discussion

### FESTA-Dried Aerogels

To produce aerogels suitable for ambient drying, the design of a robust mechanical framework and optimization of the drying process are essential. In this study, we present the fabrication of cellulose/silica nanofiber aerogels using the FESTA protocol, as illustrated in Figure 1a and Figure S1. Electrospun silica nanofibers (SNF) (*d* ≈ 372 nm, Figure S2) and TEMPO-oxidized cellulose nanofibers (CNF) (*L* ≈ 5–10 μm, *d* ≈ 10–20 nm) were first mixed and homogenized into a stable aqueous dispersion.

**Figure 1:**
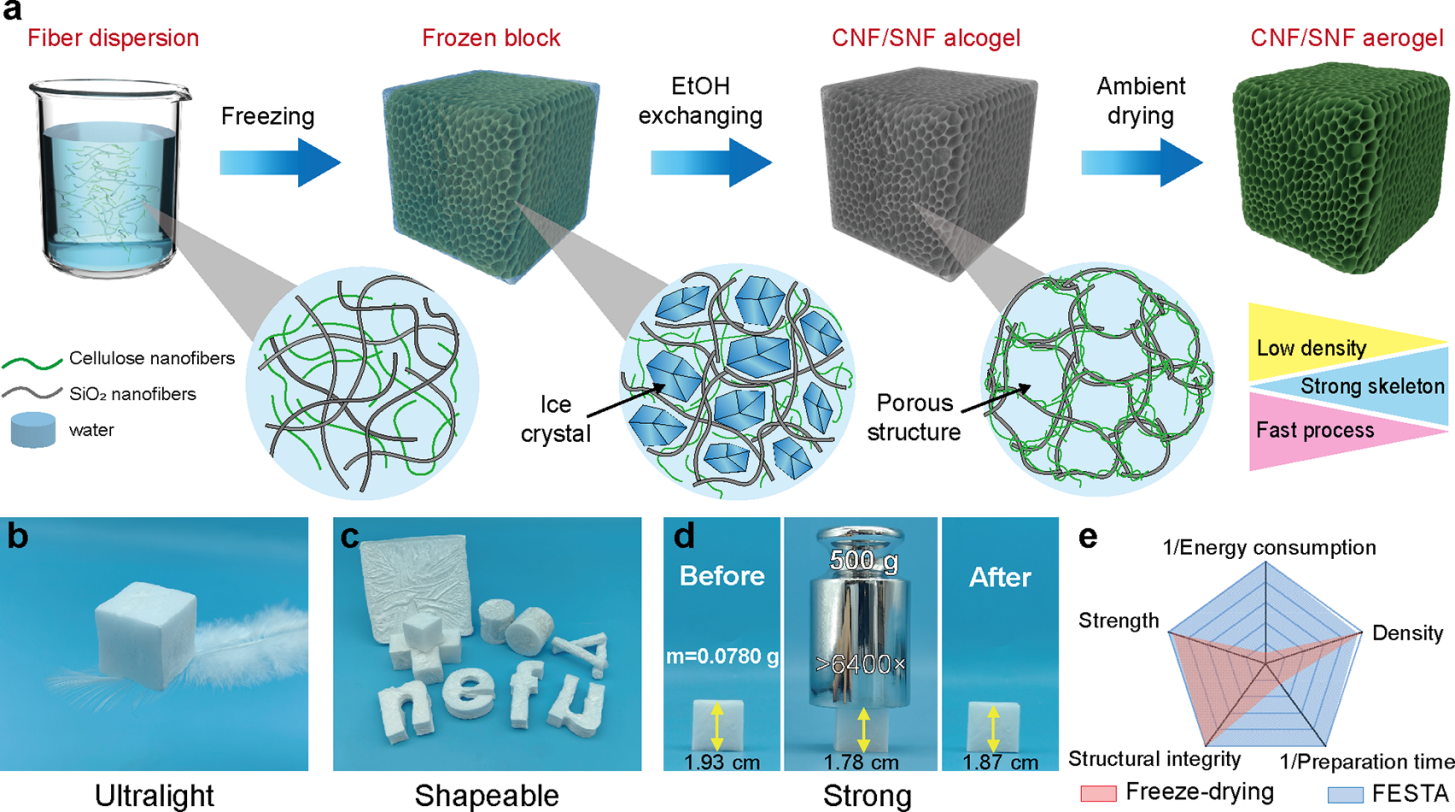
Fabrication of CNF/SNF aerogels via freezing-ethanol solvent exchange-thawing and ambient (FESTA) drying: (a) schematic illustration of the synthesis procedure. Photographs displaying some of the features of CNF/SNF aerogels: (b) ultralight weight, (c) shape adaptability, and (d) strength. (e) Qualitative radar plot showing a relative comparison of CNF/SNF aerogels synthesized by freeze-drying and FESTA, compared according to five features: energy consumption, density, mechanical strength, structural integrity, and preparation time.

Upon freezing, the CNF and SNF in the medium gradually became confined between ice crystals, forming a reinforced fibrous network through interparticle interactions. The aerogels developed mechanical strength from two synergistic factors: (i) hydrogen bonding between the cellulose nanofibers^33^ and (ii) the intrinsic structural integrity of SNFs.^34^ The resulting aerogel’s cell walls resemble reinforced concrete, where the coarse SNFs, obtained via electrospinning, act as a robust scaffold that provides overall strength and stability. These SNFs consist of an amorphous SiO_2_ network,^35^ with exceptional flexibility and mechanical strength, allowing them to withstand significant bending deformation without brittle fracture, while the finer cellulose fibrils (CNF) enhance ductility and resistance to cracking. The CNF/SNF dual-nanofiber network shows a strong cell wall structure^36^ and, at an optimized CNF-to-SNF ratio, the network effectively resists shrinkage caused by surface tension and capillary pressure during solvent exchange, thawing, and ambient drying.

The FESTA-dried CNF/SNF aerogels exhibited minimal shrinkage (8.37 ± 1.62 vol %) (Table S1) while maintaining an ultralow density (10.86 ± 0.32 mg cm^–3^), comparable to their freeze-dried counterparts (10.27 ± 0.26 mg cm^–3^). Notably, this represents one of the lowest densities ever reported for room-temperature-dried aerogels, which is particularly significant given the absence of cross-linking agents (Figure 1b).

Due to their high dimensional stability, FESTA drying enables on-demand shape molding, allowing aerogels to be fabricated into various customizable forms (Figure 1c). Beyond their shaping versatility, the FESTA-dried CNF/SNF aerogels also exhibit robust mechanical properties, demonstrating reversible compressibility. As shown in Figure 1d, a lightweight aerogel sample (0.078 g, ρ = 11.57 mg cm^–3^) successfully supports a 500 g load—approximately 6400 times its own weight—with only ∼7.8% compressive deformation. Remarkably, upon unloading, the aerogel recovers up to 96.9% of its original dimensions, highlighting its outstanding mechanical resilience.

The simplicity of the FESTA drying process also makes the aerogels highly scalable. For example, we successfully fabricated a large CNF/SNF aerogel with a total volume of approximately 500 cm^3^ (Figure S3). Compared to the widely used freeze-drying method, FESTA-dried aerogels exhibit similar structural integrity, density, and mechanical properties while significantly reducing the preparation time and energy consumption (Figure 1e).

### Morphology and Mechanical Properties

Ethanol serves as both the exchanging and evaporation solvent in the FESTA process due to its nonpolar nature, volatility, low surface tension, and eco-friendliness. These attributes collectively enable a rapid and efficient room-temperature operation, allowing a 2 × 2 × 2 cm^3^ sample to form a CNF/SNF alcogel in approximately 1.5 h (Figure 2a,**b**). The role of ethanol in the FESTA process was visually examined by placing a frozen dispersion sample in Sudan-Red-stained ethanol at room temperature. The red stain rapidly spread to the center of the frozen cube within 30 min, indicating the quick diffusion of ethanol into the ice bulk. This process is initiated at the solid–liquid interface, where ethanol preferentially displaces interfacial water before infiltrating ice crystals, ensuring gradual solvent exchange without abrupt structural disruption. Critically, ethanol’s low surface tension reduces capillary pressure by ∼69.4% compared to water during ambient drying, as derived from the Laplace equation (Discussion 1, Supporting Information). This dual functionality—efficient solvent exchange and minimized capillary stress—collectively underpins the structural fidelity of the aerogel under mild drying conditions.

**Figure 2:**
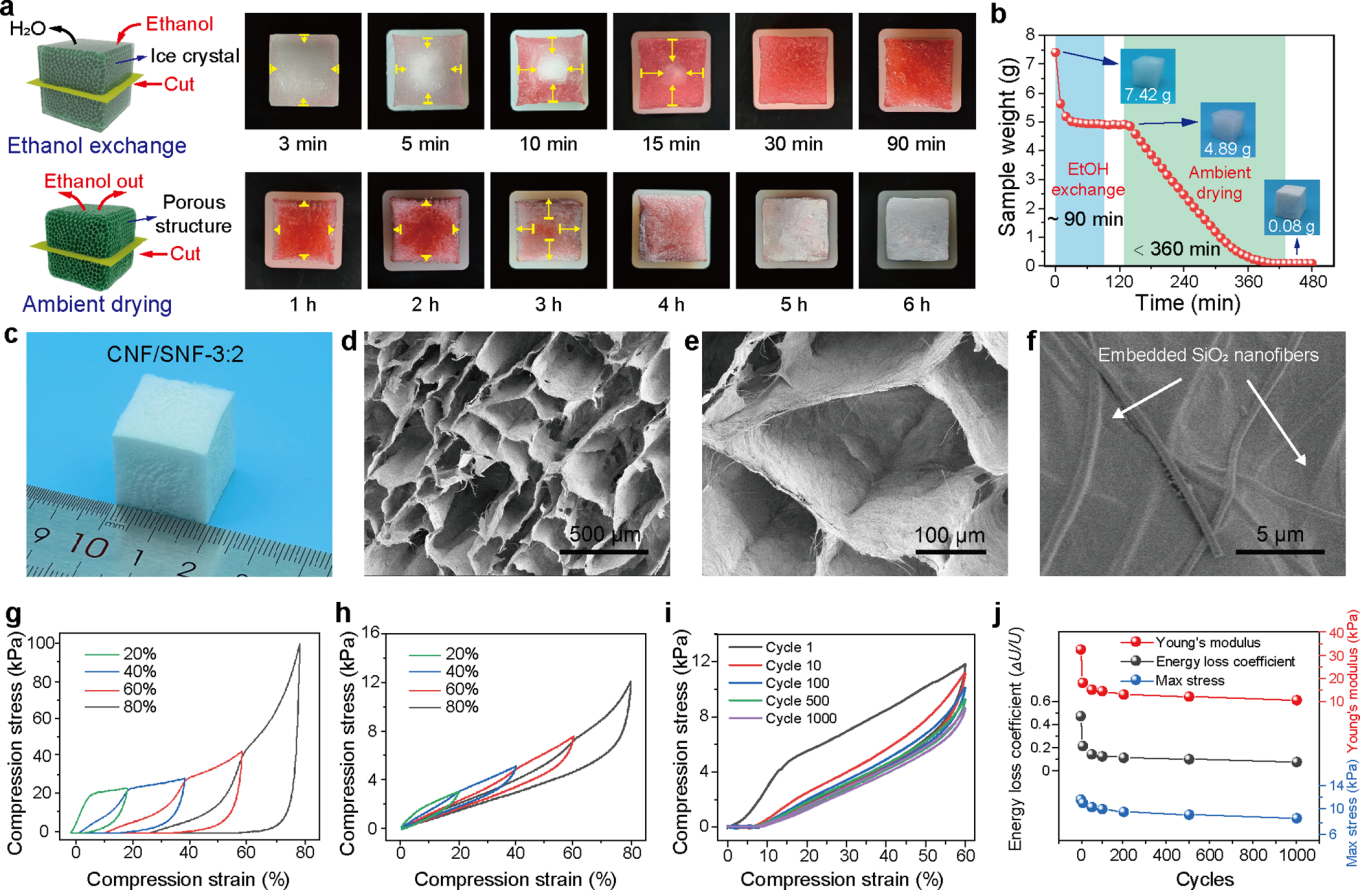
(a) Ethanol exchange and (b) ambient drying of frozen samples upon ice removal with ethanol (dyed in red color). Note that the area of samples in parts a and b is approximately 2 × 2 cm^2^. The yellow arrows indicate the depths of the transition area. (c) Mass change of samples during ethanol exchange and ambient drying. (d–f) SEM images of CNF/SNF-3:2 aerogel. (g) Stress–strain curves of CNF/SNF aerogels at various strains. (h) Underwater compression stress–strain curves of CNF/SNF aerogels. (i) Fatigue resistance at 60% strain underwater compression for 1000 cycles. (j) Young’s modulus, energy loss coefficient, and maximum stress of CNF/SNF aerogels at 60% strain during cyclic underwater compression.

A key advantage of the FESTA process is that the resulting mechanically robust aerogel does not require any cross-linking agents—neither chemical nor physical (ionic cross-linking)—reducing chemical involvement and simplifying the protocol. We note that the ambient-dried aerogels exhibited an open-cell foam structure.

Ethanol molecules promote the aggregation and assembly of CNFs,^37^ reinforcing the dual-fiber network. Moreover, ethanol’s volatility facilitates the drying process; for example, a frozen 2 × 2 × 2 cm^3^ sample achieved complete dryness within 6 h, compared to the 36–48 h typically required for freeze-drying (Figure 2c). This time efficiency is attributed to thermodynamic and kinetic effects: the thermodynamic energy barrier for FESTA (1202.9 kJ kg^–1^) is significantly lower than that of ice sublimation (2834 kJ kg^–1^) in a vacuum (see Discussion 2, Supporting Information). Moreover, under high osmotic pressure, solvent molecules penetrate wet interfaces more readily than gas molecules traverse dry, dense interfaces, as the liquid layer on wet surfaces facilitates diffusion while dry surfaces impose greater physical barriers.^38^

Nanofiber concentration plays a critical role in the successful synthesis of CNF/SNF aerogels through the FESTA method. Aerogels with a reduced density were obtained at a low nanofiber concentration in the precursor dispersions (<0.4 wt %) but at the expense of structural stability. Morphological evaluations (Figure S4) revealed that robust CNF/SNF scaffolds were easily achieved at 1 wt % precursor concentration. This condition was chosen to achieve highly porous and strong structures.

At the same solid content (1 wt %), the CNF-to-SNF ratio significantly influenced the FESTA processes, as shown by the morphological evolution of aerogels at various compositions (Figure 2d–f, Figures S5–S8). CNF is critical for maintaining the structural integrity of the aerogel; aerogels produced with SNF alone disintegrate during thawing. Those produced with neat CNF collapsed catastrophically during the thawing and drying stages, with significant volumetric shrinkage (>59.3%). Their cell walls, only ∼1.5 μm thick (Figures S6a,f), were unable to withstand capillary forces during drying.

CNF forms strong interparticle hydrogen bonding when brought together by ice templating,^39,40^ causing aggregation and localized drying stress. Increasing the SNF concentration led to more robust porous structures with thicker cell walls (Figure S6g–j), which underwent limited shrinkage. The primary role of SNFs is to disrupt the hydrogen bonding network between CNFs and form more stacked pore spaces. These larger pores, together with the thicker cell walls, are the main reasons explaining the resistance of the aerogels to evaporation-induced capillary pressure.^41^

The increased number of pores and channels enhances the rapid transport of gases and liquids, thereby expediting solvent exchange and drying.^42^ As shown in Figure S9, Brunauer–Emmett–Teller (BET) analysis revealed a strong correlation between the specific surface area (SSA) and the CNF content. An increased silica content led to a larger average pore diameter. However, excessive amounts of silica nanofibers negatively impacted the SSA due to the reduced concentration of CNF in the cell walls. An optimal aerogel formulation achieved an SSA of 104.2 m^2^/g, with a Barrett–Joyner–Halenda (BJH) average pore size of 8.1 nm. These CNF/SNF aerogels, characterized by their regular porous structure, exhibited the typical compressive mechanical behavior of open-cell foams.

The stress–strain profiles of the aerogels (Figure 2g and Figure S10) highlight three distinct stages: (i) a linear elastic region (<8% strain), corresponding to the viscoelastic bending of the cell walls; (ii) a plateau stage at intermediate strains (8 to 60%), attributed to elastic buckling; and (iii) a densification stage at high strain (>60%), where cell walls collapse and come into contact, causing a sharp increase in stress. Despite their extremely low density (∼11 mg cm^–3^), the aerogels demonstrate a high compressive strength (101 kPa) and Young’s modulus (350 kPa). These mechanical properties are attributed to physical cross-linking during the solvent exchange process^43^ and the robust porous structure, featuring large pores and thick wall layers formed after ambient drying.

The mechanical curves of the alcogel and thawed hydrogel illustrate the transformation induced by solvent exchange (Figure S11). The alcogel achieves a maximum compressive strength of 16 kPa, approximately 14.5 times greater than that of the hydrogel (1.1 kPa). Additionally, Young’s modulus increases significantly, from 3.2 kPa for the hydrogel to 156.4 kPa for the alcogel.

### Water Stability

The FESTA-dried CNF/SNF aerogels exhibited exceptional water stability—a critical property for advanced applications such as hemostatic dressings,^44^ liquid electrolytes,^45^ and hygroscopic materials.^46^ The results compared favorably against the wet stability of cellulose-based aerogels.^47^ Figure 2h and Figure S12 demonstrate the superior underwater compressibility of the aerogels, as shown in underwater compressive stress–strain (σ–ε) profiles. Moreover, the CNF/SNF aerogels endured up to 80% strain without noticeable collapse or cracking and spontaneously recovered their original shape upon unloading. During 1000 cycles of compression at 60% strain, the aerogels exhibited 7.33% plastic deformation, which indicated minimal fatigue and long-term structural integrity (Figure 2i).

As illustrated in Figure 2j, Young’s modulus, energy loss coefficient, and maximum stress decreased during the initial compression cycle but stabilized in subsequent cycles, persisting even after 1000 cycles. This stability underscores the aerogels’ excellent underwater compressive fatigue performance.

During underwater compression, the bending of nanofiber cell walls resulted in the removal of water from the pores, while the release cycle allowed the rapid re-entry of water by fast capillary action. The wet cell walls of the aerogels exhibited exceptional strength, and their highly porous structure enabled the free flow of water during deformation. This is attributed to the flexibility of CNFs and electrospun silica nanofibers, as well as the strong interfiber entanglements that reinforce the cell walls.

The wet CNF/SNF aerogels exhibited shape memory properties, likely a result of the hydrogen bond network formed during ice templating and ethanol exchange (see details in Figures S13–S16, Videos S1–S3, and Discussion 3 in the Supporting Information). During ice templating, the nucleation and growth of ice crystals initiate the formation of interfibril hydrogen bonding. The subsequent ethanol exchange disrupts these hydrogen bonds, and when ethanol evaporates, the hydrogen bonding between CNF is restored.

Upon rehydration, water molecules rapidly infiltrate the structure, dynamically reestablishing hydrogen bonds with cellulose, thereby inducing shape recovery. Remarkably, the shape recovery mechanism remains effective after the aerogel is immersed in liquid nitrogen (Figure S17): the nanofiber cell walls retain flexibility, in contrast to the brittleness of most polymer aerogels. Notably, this water-responsive shape recovery behavior is absent in aerogels fabricated via direct freeze-drying. Overall, the findings confirm that the ethanol-induced “formation–disruption–reformation” of hydrogen bonds is critical in establishing the shape memory properties of the CNF/SNF aerogels.

### Atmospheric Water Harvesting (AWH)

The porosity, ease of preparation, and wet and dimensional stability of the developed aerogels make them excellent candidates for AWH.^48^ For this purpose, we incorporated carbon nanotubes (CNTs) into the nanofibrous network during the FESTA process, resulting in robust CNT/CNF/SNF (CCS) aerogels. In this process, CNFs enhanced the colloidal dispersion of hydrophobic CNTs in water relaxing the need for dispersants.^49^

The CCS aerogels were further immersed in an aqueous solution of the hygroscopic salt lithium chloride (Figure 3a). The resulting hybrid aerogel, referred to as LiCl@CCS, exhibited high porosity, suitable mechanical properties, and superior moisture sorption capability across a wide humidity range. The morphology of the LiCl@CCS aerogel is advantageous for capturing ambient moisture. The cellular pore structure facilitates rapid moisture transfer at the absorbent–air interface. Scanning electron microscopy (SEM) images (Figure 3b) reveal small, uniform pores measuring 200–300 μm, formed during ice templating. The surfaces of the cell walls are rough and present intertwined SNF, CNT, and CNF, resulting in a stable and robust network.

**Figure 3:**
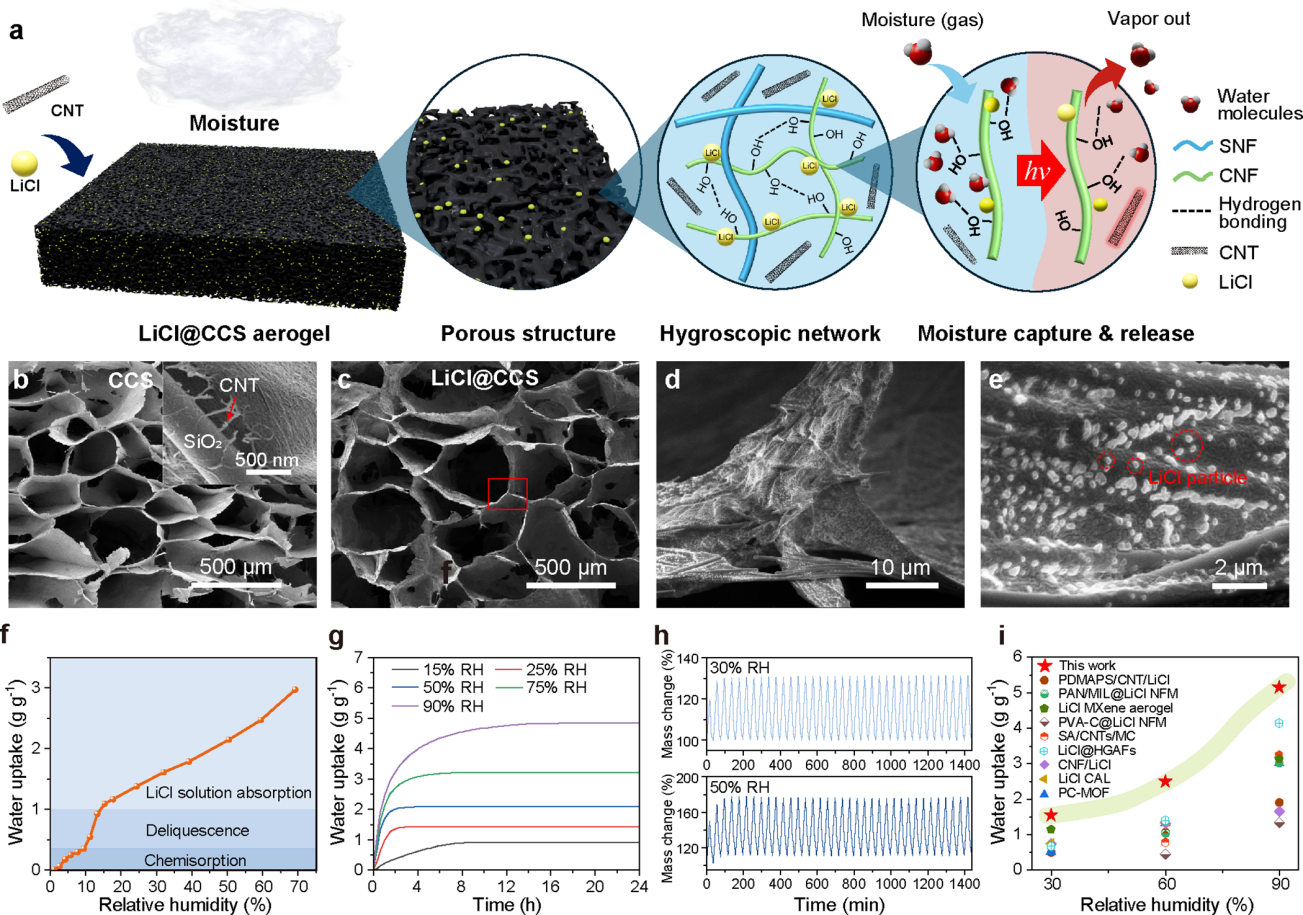
Solar-driven CNF/SNF aerogel-based AWH device. (a) Schematic illustration of the design principle. SEM images of (b) CCS and (c–e) LiCl@CCS aerogels. The SEM images indicate the embedding of CNT and LiCl in the porous matrix. (f) Vapor sorption isotherm of LiCl@CCS aerogels. (g) Dynamic water vapor sorption curves of the LiCl@CCS aerogels at 15, 30, 50, 75, and 90% RH. (h) Cycling performance of LiCl@CCS aerogels, where sorption occurs at 30% RH and 50% RH (20 min per cycle, 36 cycles per day). The desorption is performed at 80 °C for 20 min per cycle. (i) Comparison of moisture sorption capacity for some reported hygroscopic materials and compared to results from this study (star symbol).

Higher magnification SEM imaging and X-ray diffraction (XRD) analysis confirm the successful incorporation of lithium chloride (Figure 3d,e and Figure S20). LiCl crystals were uniformly distributed across the cell wall surfaces without significant aggregation. This uniform distribution not only prevents salt leakage during operation but also maintains the functionality of the system during SAW cycling.

The aerogel’s porous structure increases the active surface area available for LiCl to interact with moisture, while simultaneously enabling rapid water vapor transmission and water storage capacity. Even under high-humidity conditions (80–90% RH), continuous moisture uptake is sustained, as water is confined and retained within the porous framework via capillary forces, effectively preventing leakage and overflow (Figure S21). In sum, the developed LiCl@CCS aerogels are expected to maintain excellent performance for moisture capture in diverse environmental conditions.

In AWH, hygroscopic materials must release absorbed water under the given conditions. Solar-driven desorption is an ideal strategy to complete the water harvesting cycle for aerogel sorbents due to its sustainability, availability, and environmental benefits.^50^ This approach is particularly advantageous in arid regions, which experience consistent and intense daylight.

In this work, carbon nanotubes (CNTs) effectively convert sunlight into thermal energy, facilitating the rapid evaporation of sorbed moisture and enabling a stable cycle of water sorption and desorption. CNF integration not only stabilizes the composite structure but also promotes rapid water transfer, allowing for efficient capture and release of water by LiCl.

Dynamic vapor sorption (DVS) was measured under constant airflow. Humid water vapor diffuses through the open pores of the aerogel to the cell wall surface due to the vapor pressure difference. Lithium chloride (LiCl) plays a critical role in the water sorption capacity of the LiCl@CCS aerogels from humid air at 25 °C, shown to occur in three distinct stages (Figure 3f):

1. Below the deliquescence RH (∼11%): Water uptake is driven by the interaction of anhydrous LiCl with water molecules.
2. Intermediate humidity range (11–15% RH): Additional water is absorbed at the liquid–gas interface, resulting in the deliquescence of hydrous lithium chloride (LiCl·H_2_O) until saturation.
3. Higher humidity (>15% RH): Water vapor sorption occurs in saturated or concentrated LiCl system, significantly contributing to hygroscopicity.

During the sorption phase, excess water is stored in the cellulose network facilitated by the hydrophilic hydroxyl groups. LiCl provides the necessary hygroscopicity for high water sorption, while the aerogel’s high porosity enhances diffusion dynamics and serves as a reservoir for water uptake.

The LiCl loading in the aerogel is crucial for optimizing the moisture sorption capacity. The efficiency of water uptake was evaluated at different LiCl concentrations at 25 °C (Figure S22). A minimum LiCl concentration of 4% (w/v) is required for a significant water uptake. However, excessive loading leads to aggregation and uneven distribution of LiCl salts, increasing the risk of water leakage after sorption. A suitable LiCl concentration was determined to be 6% (w/v) in the secondary immersion solution.

The optimized LiCl@CCS aerogels demonstrate excellent environmental adaptability and water uptake performance across a range of humidity conditions. At relative humidities (RH) of 15, 25, 50, and 75%, the aerogel achieves water uptake of 0.90, 1.43, 2.09, and 3.21 g g^–1^, respectively, outperforming most reported moisture sorption materials (Figure 3g,i).^51–59^ Within the 25–75% RH range, the aerogel reaches equilibrium within 6 h, reflecting favorable sorption dynamics. Additionally, under extremely humid conditions (90% RH), the LiCl@CCS aerogel absorbs over 4.85 g g^–1^ water after reaching saturation. Notably, despite the incorporation of hydrophobic CNTs, the overall hygroscopic performance remains comparable to that of the CNT-free sample, suggesting minimal impact on the water sorption capacity (Figures S23 and 24).

To investigate the desorption efficiency, we subjected the water-saturated LiCl@CCS aerogel to a closed chamber at 80 °C, simulating material surface temperatures under sunlight. As shown in Figure S26, the aerogel exhibited rapid desorption, with a sharp decline in mass within the first 10 min, corresponding to water release. Equilibrium was reached within 30 min, highlighting the rapid desorption. However, practical desorption conditions depend on the environmental humidity and thermal diffusion rates. To simulate more realistic conditions, a lower evaporation temperature of 60 °C was selected. Sorption–desorption cycles were conducted for 36 cycles per day at 30% RH and 50% RH (Figure 3h). After 36 cycles, the aerogel maintained stable sorption and desorption efficiency without degradation or LiCl leakage, confirming its durability and dynamic performance. Moreover, this stability was not limited to low-humidity conditions; even under 10 repeated sorption–desorption cycles at high relative humidity (90% RH), the aerogel continued to exhibit reliable and consistent performance (Figure S27).

### Photothermal Conversion during Atmospheric Water Harvesting

The photothermal conversion of the LiCl@CCS aerogel was evaluated by using a solar simulator at an irradiation intensity of 1.0 kW m^–2^. Under the standard solar spectrum (AM1.5 G), the aerogel achieved a solar absorption rate of 97.4% across the 250–2500 nm wavelength range, significantly higher than the 61.7% of LiCl@CS (Figure 4a). When exposed to simulated sunlight, the surface temperature of the LiCl@CCS aerogel rapidly increased to 55.7 °C within 5 min and 84.4 °C within 60 min, compared to only 31.3 and 38.4 °C for the LiCl@CS aerogel (Figure 4b,c). This enhanced photothermal performance is attributed to the carbon nanotubes (CNTs) that introduce photothermal conversion.^60^

**Figure 4:**
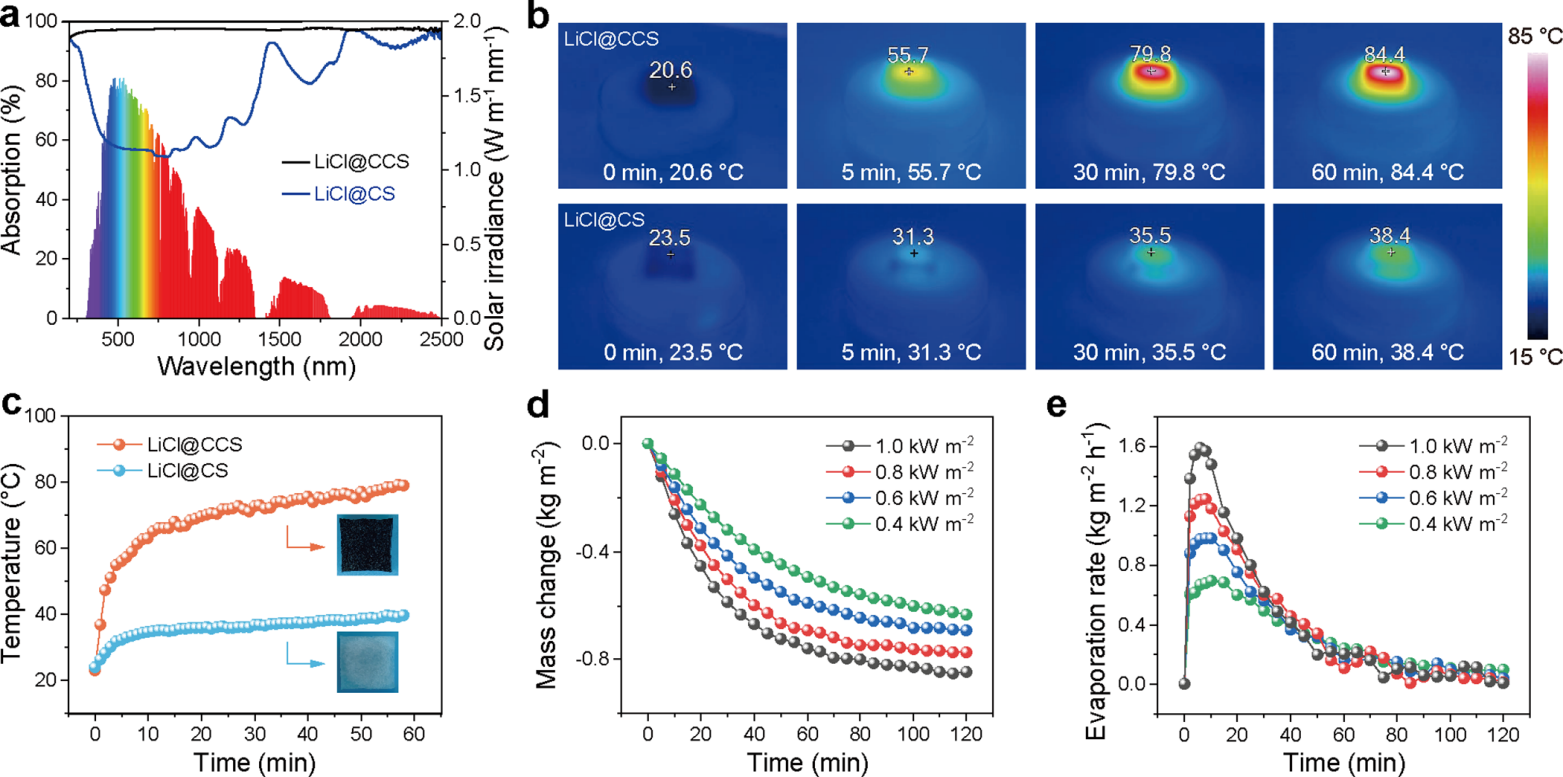
Characterization of solar-driven photothermal and water release performance of LiCl@CCS aerogels. (a) Solar spectrum and UV–vis–NIR absorption spectra of LiCl@CS and LiCl@CCS aerogels. (b) IR images and surface temperature evolution profiles of LiCl@CCS aerogel under one-sun irradiation from 0 to 60 min. (d) Mass change and (e) evaporation rate of LiCl@CCS aerogels under various solar irradiation areal intensities.

The intensity of solar radiation varies depending on factors such as latitude, season, weather conditions, and time of day, and it does not always correspond to a high light intensity. Consequently, it is valuable to assess the performance under low lighting. The evaporation performance of the LiCl@CCS aerogel was assessed by measuring cumulative mass losses under simulated sunlight irradiation ranging from 0.4 to 1.0 kW m^–2^.

As shown in Figure 4d,e, the mass loss followed a nearly linear trend during the first 10 min, after which the evaporation rate decreased and stabilized. Under one-sun illumination (1.0 kW m^–2^), the maximum evaporation rate of the LiCl@CCS aerogel was calculated at 1.59 kg m^–2^ h^–1^. Even at low irradiation levels of 0.4 sun illumination, the aerogel achieved a good evaporation rate of 0.69 kg m^–2^ h^–1^.

These results confirm that the LiCl@CCS aerogel effectively operates across a broad range of sunlight intensities. The rapid evaporation rate, even under low-light conditions, can be attributed to its excellent light absorption, efficient photothermal conversion, moisture permeability, and rapid moisture transfer. Finally, the LiCl@CCS aerogel demonstrated resilience against the capillary forces generated during liquid evaporation, maintaining its structural integrity without dimensional instability throughout the evaporation process.

### Water Collection in Outdoor Conditions

Considering that the ultimate goal of atmospheric water harvesting is to obtain a condensate, we evaluated the freshwater productivity of LiCl@CCS aerogels in outdoor conditions. The experiments were conducted by using a custom-designed condensate collection apparatus. The process consisted of two distinct stages: nighttime moisture absorption and daytime desorption.

During the nighttime absorption stage, the aerogels were exposed to atmospheric humid air, allowing them to fully sorb and store water vapor. In the daytime desorption stage, the aerogels were placed under outdoor sunlight (Figure 5a) within a semiclosed container designed to enhance condensate collection. The photothermal properties of the LiCl@CCS aerogel enabled surface heating under sunlight, triggering the release of sorbed water.

**Figure 5:**
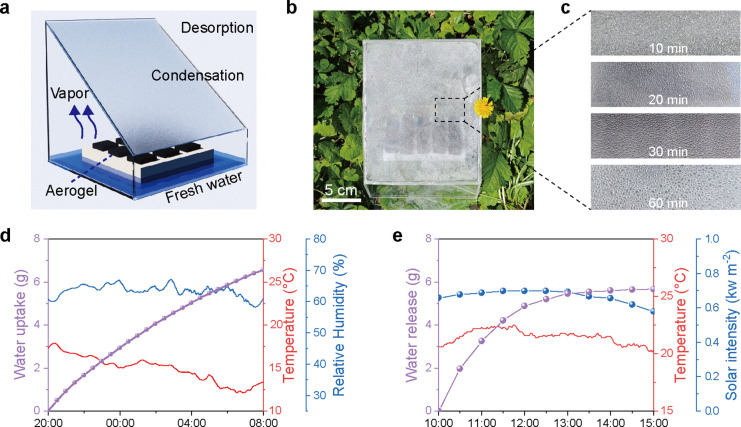
Outdoor water collection experiment conducted on September 26–27, 2024, in Harbin, China. (a) Schematic illustration of the structure of an AWH device based on LiCl@CCS aerogel. (b) Photograph showing the top surface of the device after solar illumination. (c) Optical photograph showing the condensed water droplets on the wall of the device after various times of solar illumination. (d) Temperature, relative humidity, and the water uptake at night in the adsorption process. (e) Temperature, solar intensity, and water release during the day in the desorption process.

The released water vapor contacted the plastic sidewalls of the container, which were cooled by a breeze. This cooling effect facilitated the condensation of water vapor into small droplets. These droplets gradually grew, coalesced, rolled down under gravity, and ultimately collected as liquid water (Figure 5b,c).

The LiCl@CCS aerogels demonstrated a consistent increase in mass during a 12 h nighttime moisture absorption period at a relative humidity of 50–70% RH. Specifically, 3.50 g of aerogel absorbed 6.57 g of water, corresponding to a water uptake of 1.88 g g^–1^ (Figure 5d). As the process progressed, the rate of water sorption gradually decreased as the LiCl solution approached saturation.

Following the water sorption phase, the device was sealed, and desorption experiments were initiated under outdoor sunlight starting at 10 a.m. The sunlight intensity ranged from 0.5–0.7 kW m^–2^, with an ambient temperature of approximately 22 °C (Figure 5e). The increasing light intensity in the morning facilitated rapid desorption from the LiCl@CCS aerogels, resulting in a significant weight loss and water release.

As the water vapor accumulated, a higher partial pressure developed within the container, driving condensation on the cooled sidewalls and the top cover of the apparatus. This condensation was enabled by the temperature gradient between the device’s interior and the ambient atmosphere, promoting efficient convective heat transfer. In contrast, desorption occurred after 2 p.m. under reduced light when most of the water had already been desorbed.

After desorption for 5 h, the aerogels released a total of 5.67 g of water, with 4.10 g collected as a liquid condensate. This corresponds to a water collection efficiency of 62.3% and a water production rate of 1.17 g g^–1^ day^–1^. Furthermore, the aerogels maintained a stable and reliable water harvesting performance during continuous outdoor tests under similarly low solar intensity conditions (Figure S28). These results are particularly noteworthy given the relatively low solar intensity, underscoring the applicability of LiCl@CCS aerogels for clean water collection under low-light conditions.

## Conclusions

In this study, we used freezing-ethanol solvent exchange-thawing-ambient (FESTA) drying to produce aerogels with outstanding performance in atmospheric moisture harvesting (AWH), enabling rapid and stable water production. The aerogel structure, formed by a dual-nanofiber network of cellulose and silica nanofibers, provided mutual reinforcement, allowing the aerogels to withstand capillary forces during drying without the need for cross-linking agents.

The FESTA drying process significantly reduced preparation time by over 80% compared to traditional freeze-drying methods, while maintaining a balance between ultralow density (10.86 ± 0.32 mg cm^–3^) and favorable mechanical properties (Young’s modulus: 350 kPa). This advancement offers a scalable and energy-efficient alternative to conventional drying techniques and opens the development of novel ambient-dried aerogel compositions.

Furthermore, the aerogels were easily functionalized with lithium chloride and carbon nanotubes for hygroscopic water sorption and photothermal conversion. The functionalized aerogel composite (LiCl@CCS) demonstrated stable water absorption and evaporation rates, achieving a daily water production of 1.17 g g^–1^ under outdoor conditions. These findings mark a significant breakthrough in ambient-dried aerogels, highlighting their potential for sustainable and efficient atmospheric moisture harvesting applications.

## Experimental Section

### Chemical and Materials

Cellulose nanofibril suspension was obtained from Tianjin Woodelf Biotechnology Co., Ltd. (Tianjin, China). These cellulose nanofibrils were produced from bleached hardwood kraft pulp via TEMPO-mediated oxidation, characterized by lengths of approximately 5–10 μm and diameters ranging from 10 to 20 nm, with a carboxylate content of around 1.4 mmol/g. Tetraethyl orthosilicate (TEOS), poly­(vinyl alcohol) (PVA, Mw = 88 000, hydrolyzing degree of 88–89% mol mol^–1^), phosphoric acid, ethanol (AR grade, 99.7%), and lithium chloride (LiCl, ≥99%) were acquired from Aladdin Reagent (Shanghai, China). Multiwall carbon nanotubes (MWCNT, > 95%, 10–30 μm in length and 5–15 nm in diameter) were purchased from Xianfeng Nanomaterial Technology Co. Ltd. (Nanjing, China). All reagents were of analytical grade and utilized as received, without any further purification.

### Preparation of CNF/SNF Aerogel

First, according to previous studies, the SiO_2_ nanofibers were synthesized by the calcination of the electrospinning silica precursor.^36^ Then, SiO_2_ nanofibers were cut into small sheets and homogenized by a high-speed homogenizer (IKA Ultra-Turrax Homogenizer) at high speed (10000 rpm) for 10 min. The SiO_2_ nanofibers dispersion was then filtered to remove water and dried to obtain a SiO_2_ nanofibrous mat for further use. To prepare the hybrid nanofibrous suspensions, SiO_2_ nanofibrous (SNF) mats were first incorporated into cellulose nanofibers dispersion with various weight ratios of 0.2, 0.4, 0.6, and 0.8 wt %, until the total solid content reached 1.0 wt %. The mixture was subjected to ultrasonic dispersion using a high-intensity ultrasonicator set at an output power of 800 W for 20 min, resulting in a homogeneous CNF/SNF nanofibers suspension. Subsequently, the obtained suspension was cast into soft silicone molds and frozen in a refrigerator at −20 °C. After completely freezing, the blocks were removed from the molds and immersed in ethanol (at a volume 10 times that of the sample) at ambient temperature to facilitate the displacement into CNF/SNF alcogels. Once fully thawed, the CNF/SNF alcogels were withdrawn from the ethanol and dried by ambient drying.

### Preparation of LiCl@CCS Aerogel

The fabrication of LiCl@CNT/CNF/SNF (LiCl@CCS) aerogel was conducted similarly to the process for atmospheric drying aerogels, but incorporating 0.2 wt % multiwalled carbon nanotubes (CNT) into the CNF/SNF nanofiber suspension. This mixture was then subjected to freezing at −20 °C, followed by ethanol displacement and atmospheric drying. To prepare CNF/SNF/CNT@LiCl aerogel at various LiCl loadings, CNF/SNF/CNT aerogels were immersed in LiCl with different concentrations (2, 4, 6, 8, and 10 g of anhydrous lithium chloride were dissolved in 100 mL of 90% ethanol solution, respectively). Vacuum was applied to facilitate the complete penetration of the lithium chloride solution into the aerogel’s structure. Finally, the aerogel was dried at 80 °C to obtain a hygroscopic CNF/SNF/CNT@LiCl system.

### Characterization

The morphologies and structures of the aerogels were investigated by using scanning electron microscopy (SEM, FEI Apreos). The FTIR spectra were collected by using a Fourier transform infrared spectrometer (FTIR, Nicolet iN10, Thermo Fisher Scientific Inc., USA).

The mechanical properties of the aerogel samples (20 × 20 × 20 mm^3^) were measured using the universal testing machine (Suns, UTM2503, China) for compression tests, including compression in air, elasticity, and fatigue resistance under water.

The specific surface area and pore sizes of the aerogels were measured with a specific surface analyzer (BET, BSD-PS, China) at 77.3 K.

X-ray diffraction (XRD) patterns were analyzed with a XRD Diffractometer (PANalytical Empyrean) with an angular range of 10–90° (2 theta).

The light absorption spectra of samples were measured by the UV–vis-NIR spectrophotometer (UV-3600i Plus, Shimadzu, Japan) in the range of 250 to 2500 nm with an integrating sphere.

Dynamic water vapor sorption–desorption experiments were measured by a dynamic vapor sorption (DVS) instrument (DVS Adventure, Surface Measurement Systems LTD, Ltd.) at 25 °C. Before the measurement, all aerogel samples (10 × 10 × 5 mm^3^) were preheated to dry at 100 °C and 0% RH for 4 h to be completely dehydrated before the sorption tests.

Thermographic images were captured using an infrared (IR) thermal camera (Testo 869, Testo AG, Germany), while temperature–time curves were recorded with a multichannel thermocouple (VC8801–16, Victor, China).

Photothermal desorption experiments were performed with a solar simulator (CEL-S500, Ceaulight, China) equipped with an AM 1.5G solar filter to replicate natural sunlight conditions. The intensity of the simulated solar irradiation was measured and calibrated from 0.4 to 1.0 kW m^–2^ using an optical power meter (CEL-FZ-A, China). The mass change of the samples was monitored using an electronic balance (0.1 mg in accuracy, CP214, OHAUS, China), and the sample sizes were 20 × 20 × 5 mm^3^.

The outdoor water harvesting experiment was conducted in an open area in Harbin (45.72°N, 126.63°E). The solar intensity was recorded using an optical power meter (CEL-FZ-A, China), while the samples’ surface as well as that of the ambient were recorded (VC8801–16, China). The ambient humidity was monitored with a humidity Meter (MS6508, China).

## Supplementary Material









## Notes

Experimental details and supporting data mentioned in the paper, including the calculation of capillary pressure and energy consumption; experimental details; characterization of SNF; large-sized aerogel; demonstrations of CNF/SNF aerogels with varying nanofibers and CNF contents; morphology of CNF/SNF aerogels; density, volume shrinkage and porosity of CNF/SNF aerogels; mechanical properties; FTIR spectra; wet and chemical stability; XRD patterns; high-humidity stability test; water uptake tests with various LiCl loadings; stepwise moisture adsorption curves; water contact angle of CCS aerogels; relationship between moisture absorption/desorption behavior and LiCl@CCS sizes; stability during cyclic moisture absorption and desorption; continuous outdoor water harvesting experiments (PDF)
Video S1: Underwater compressive deformation and recovery of aerogels (MP4)
Video S2: Rapid shape recovery of CNF/SNF aerogels upon hydration (MP4)
Video S3: Elastic response of CNF/SNF aerogels immersed in liquid nitrogen (MP4)
