# Supplementary material for: Hybrid Nanofiber-Based Atmospheric Water Harvesters: Sunlight-Driven Operation in Low-Humidity and Low-Illumination Environments
Source: ACS Nano. 2025 May 27;19(22):20881–91. doi: 10.1021/acsnano.5c03322 (PMC13102321; doi:10.1021/acsnano.5c03322)
Supplement: Supplementary file 1 [file nn5c03322_si_001.pdf]

Supporting Information for

Hybrid Nanofiber-Based Atmospheric Water Harvesters: Sunlight-Driven Operation in Low-Humidity and Low-Illumination Environments

Yi Hu,[1,2] Yu Chen,[1] Jianing Xu,[1] Wanli Cheng,[*,1] Yi Lu,[*,2,3,4] Guangping Han,[*,1] and Orlando J. Rojas[*,2,3,5,6,7]

[1] Key Laboratory of Bio-Based Material Science and Technology (Ministry of Education), Northeast Forestry University, Harbin 150040, P.R. China

[2] Bioproducts Institute, University of British Columbia, 2385 East Mall, Vancouver, BC V6T 1Z4, Canada.

[3] Department of Chemical and Biological Engineering, University of British Columbia, 2360 East Mall, Vancouver, BC V6T 1Z3, Canada.

[4] Institute of Process Engineering, Chinese Academy of Sciences, Beijing 100190, PR China

[5] Department of Wood Science, The University of British Columbia, 2424 Main Mall #2900, Vancouver, BC V6T 1Z4, Canada

[6] Department of Chemistry, The University of British Columbia, 2036 Main Mall, Vancouver, BC V6T 1Z1, Canada

[7] Department of Bioproducts and Biosystems, Aalto University, Vuorimiehentie 1, P.O. Box 16300, 02150 Espoo, Finland

Corresponding Authors: guangping.han@nefu.edu.cn (G. Han)**,** nefucwl@nefu.edu.cn (W. Cheng)**;** luyi@ipe.ac.cn (Y. Lu), orlando.rojas@ubc.ca, orlando.rojas@aalto.fi (O. J. Rojas)

Supporting Information Content:

Supplementary Discussions 1-3

Supplementary Figures S1-S28

Supplementary Tables S1-S2

Videos S1-S3

Supplementary Discussions

Discussion 1: Calculation of capillary pressure during evaporation.

During the ambient drying process, the capillary pressure in the pore structure is the main cause of shrinkage. The capillary force is mainly derived from the surface tension of the liquid in the porous network and can be approximated by the Laplace equation:

$$P = \frac{2\gamma\cos\theta}{r}$$

where r is the pore radius, γ is the surface tension of the liquid, and θ is the contact angle between the liquid and the pore wall.

For hydrophilic aerogels, $\theta \approx 0°$ for both water and ethanol ($\cos\theta \approx 1$), meaning capillary pressure (P) is solely determined by surface tension (γ) and pore radius.

At 25 °C, water's high surface tension (γ_{water} = 72 mN/m) arises from strong intermolecular hydrogen bonding, significantly exceeding that of ethanol ($\gamma_{ethanol}$ = 22 mN/m) and other common ambient-drying solvents (e.g., acetone: 23.0 mN/m, isopropanol: 21.4 mN/m, hexane: 18.4 mN/m).

As shown in Figure S7, the CNF/SNF aerogels exhibit a pore diameter distribution centered ~300 μm (r = 150 μm). Using the above equation, the capillary pressures for water and ethanol evaporation are $P_{water} \approx$ 960 Pa, $P_{ethanol} \approx$ 293.3 Pa.

Switching from water to ethanol reduces P by a factor of ~ 3.3. Therefore, the use of low-γ solvents thus provides a practical strategy to preserve nanostructured porosity. In addition, larger pore structure can also reduce the capillary pressure in evaporation, so from the perspective of ambient pressure drying, larger pore structure and lower surface tension

are two important development paths to reduce the capillary pressure, without the need for freeze drying or supercritical drying without freeze-drying or supercritical drying.

Discussion 2: Energy consumption calculation of freeze-drying and ambient drying process.

Freezing process

For both freeze-drying and ambient drying, the samples were first frozen at room temperature to -20 ° C. The freezing process can be divided into three distinct stages, each involving different types of energy changes: sensible heat and latent heat. The steps are as follows:

$$
\begin{aligned}
Q_{total} &= Q_{cooling1} + Q_{freezing} + Q_{cooling2} \\
&= m \cdot c_{water} \cdot \Delta T_1 + m \cdot L_f + m \cdot c_{ice} \cdot \Delta T_2 \\
&= m \cdot 4.18 \cdot 25\ kJ/kg + m \cdot 334\ kJ/kg + m \cdot 2.1 \cdot 20\ kJ/kg \\
&= m \cdot 480.5\ kJ/kg
\end{aligned}
$$

where $Q_{cooling1}$ is energy consumption of cooling water from 25 °C to 0 °C, $Q_{freezing}$ is the step involves a phase change of water into ice and $Q_{cooling2}$ is the removed sensible heat from the ice as it cools from 0 °C to -20 °C, further, m is mass of the water, ΔT_1 is the temperature change from 25 °C to 0 °C, L_f is the latent heat of fusion for water (334 kJ/kg), c_{ice} is the specific heat capacity of ice (~2.1 kJ/(kg·°C)) and ΔT_2 is the temperature change from 0 °C to -20 °C.

Freeze drying

For freeze-drying, energy consumption could be roughly equal to the enthalpy change of sublimation, where ice transitions directly from the solid phase to the vapor phase, consuming sublimation latent heat. The energy required for this process can be calculated as:

$$Q_{\text{sublimation}} = m \cdot L_{sub} = m \cdot (\Delta H_{fusion} + \Delta H_{evaporization}) = m \cdot 2834\ kJ/kg$$
$$= m \cdot (334kJ/kg + 2500kJ/kg) = m \cdot 2834\ kJ/kg$$

where m is the mass of the ice, L_{sub} is the sublimation latent heat of ice, ΔH_{fusion} is the fusion latent heat and $\Delta H_{evaporization}$ is the evaporization latent heat at 0°C and 1 atm.

Ambient drying

For ambient drying, the preparation process involves thawing, solvent exchange and ambient drying (ethanol evaporation). According to Hess's law. The energy required for this process can be calculated as:

$$Q_{total} = Q_{thawing} + Q_{heating} + Q_{evaporation}$$
$$= (m_{ice} \cdot c_{ice} \cdot \Delta T_1 + m_{ice} \cdot L_f) + m_{water} \cdot c_{water} \cdot \Delta T_2 + m_{ethanol} \cdot L_{vap}$$
$$= m_{ice} \cdot 1202.9\ kJ/kg$$

Where $Q_{thawing}$ involves heating ice to 0 °C and melting ice to liquid water, $Q_{heating}$ involves heating water to 25 °C and $Q_{evaporation}$ is energy consumption for ethanol evaporation. Further, m_{ice} is the mass of ice, c_{ice} is the specific heat capacity of ice (~2.1 kJ/(kg·°C)), ΔT_1 is the temperature change from -20°C to 0 °C, L_f is the latent heat of fusion for ice (~334 kJ/kg), c_{water} is the specific heat capacity of ice (4.18 kJ/(kg·°C)), ΔT_2 is the temperature change from 0 °C to 25 °C and L_{vap} is the latent heat of vaporization for ethanol (840 kJ/kg).

Discussion 3: Shape-recovery and characterization of the CNF/SNF aerogels.

Negative Poisson's ratio

Notably, the hydrated CNF/SNF aerogels exhibited auxetic behavior (negative Poisson's ratio) during compression, as shown in the photographs in Figure S13 and Video S1. This behavior contrasts with conventional hydrated 3D closed-cell polymer materials, which typically exhibit a positive Poisson's ratio. Microscopic images (Figure S14) reveal that the porous structure favors inward buckling during compression, preserving the integrity of most cell walls. This inward buckling promotes lateral expansion, resulting in the observed zero-to-negative Poisson's ratio effect.

Shape memory and recovery

As illustrated in Figure S15, the dried aerogels were first flattened and folded, showing no shape recovery in air. However, upon immersion in water, the rapid hydration of the CNF/SNF aerogel efficiently restored its original shape. This water-induced shape memory is attributed to the capillary flow of water into the internal cellular channels. Remarkably, the entire shape recovery process took less than 1.4 seconds. Furthermore, the compressed and folded hydrated aerogel demonstrated rapid recoverability after rehydration, as shown in Video S2. This shape memory and recovery mechanism is primarily driven by the rapid capillary siphoning of water through the porous cellular channels, coupled with cellulose-induced rehydration. Given the exceptionally high porosity of the aerogel, the capillary siphoning of water emerges as the critical factor, with the small cellular pores facilitating a rapid capillary process.

When the sample was folded, the internal cellular channels were compressed and deformed, yet they did not collapse (Figure S14). The deformation of shape was stabilized by robust hydrogen bonds. During the hydration process, water infiltrating the channels first disrupted these hydrogen bonds and rehydrated the cellulose nanofibers, thereby unlocking the previously constrained hydrogen bond network and structural integrity. Furthermore, the elasticity of the aerogel during the siphoning process significantly enhances the efficiency of this capillary action.

High-resolution photomicrograph images of the nanofibrous cell wall reveal the open-pore structure and intricate architecture of the aerogel (Figure S16). The silica nanofibers (SNFs) are primarily embedded within the TEMPO-oxidized cellulose nanofiber coating layer.

Benefiting from its excellent reversible compressibility and rapid water absorption and expansion capabilities, the hydrated CNF/SNF aerogel demonstrates remarkable shape memory properties in the wet state. Notably, this aerogel retains its elasticity even at extremely low temperatures, such as immersion in liquid nitrogen at -196 °C. Unlike most polymer aerogels, which become brittle under similar conditions, the nanofibrous cell walls of the CNF/SNF aerogel remain flexible (Figure S16 and Video S3).

The chemical composition

The chemical composition of the CNF/SNF aerogels was further analyzed using Fourier Transform Infrared (FTIR) spectroscopy (Figure S18), confirming the characteristic peaks of TEMPO-oxidized cellulose (C=O at ~1605 cm^{-1}) and silica (Si-O-

Si at ~800 cm^{-1}). Importantly, no new peaks were observed, indicating that the mechanical strength arises solely from the physical entanglement of nanofibers.

Regarding the changes in the hydroxyl vibration peak, after the introduction of SNF, the hydroxyl peak at 3330 cm^{-1} shifts to 3340 cm^{-1} (Figure S18b). Since pure SNF exhibits no hydroxyl peak, the blue shift of the hydroxyl (-OH) stretching vibration in cellulose suggests a weakening of hydrogen bonding (as the vibration frequency of free hydroxyl groups is higher than that of hydrogen-bonded hydroxyls). Additionally, the significant reduction in hydroxyl peak intensity after introducing SNF may be attributed to the formation of new hydrogen bonds between cellulose hydroxyl groups and the SNF surface. This interaction likely restricts some hydroxyl vibrations and disrupts part of the intercellular hydrogen bonding in cellulose, leading to a decrease in hydrogen bond quantity.

The FTIR results, combined with the structural analysis, confirm that the mechanical integrity of the FESTA-dried CNF/SNF aerogels is attributed to the effective encapsulation of the CNF layer around the SNFs, without the need for chemical crosslinking.

Wet and chemical stability

Beyond their underwater stability, the CNF/SNF aerogels also exhibit exceptional resistance to solvents, benefiting from the inherent properties of cellulose and silica. To evaluate their morphological stability, the aerogels were immersed for two months in water, acidic and alkaline solutions, as well as common organic solvents (Figure S19). The aerogels retained their shape without any signs of disintegration or collapse, demonstrating outstanding chemical stability and solvent resistance.

Supplementary Figures

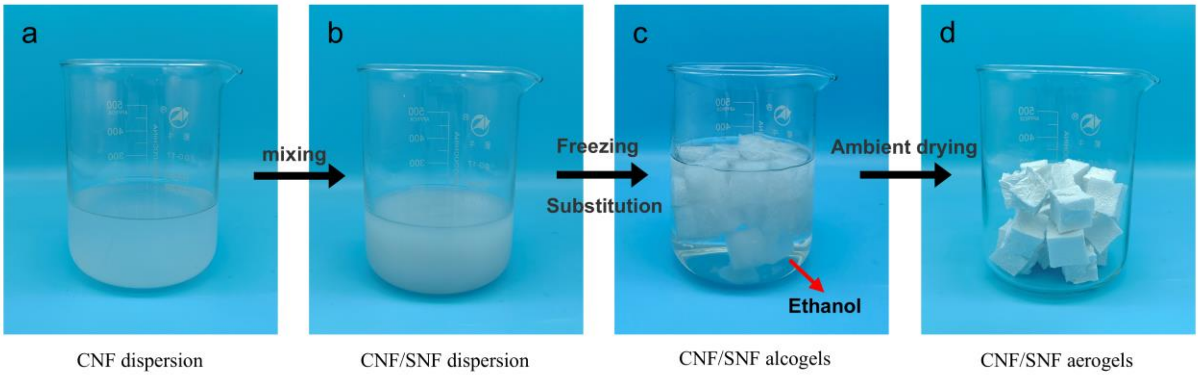

Figure S1. Photographs showing the synthesis of CNF/SNF aerogels: (a) CNF dispersion, (b) CNF/SNF dispersion, (c) CNF/SNF alcogels after ethanol exchange, and (d) CNF/SNF aerogels.

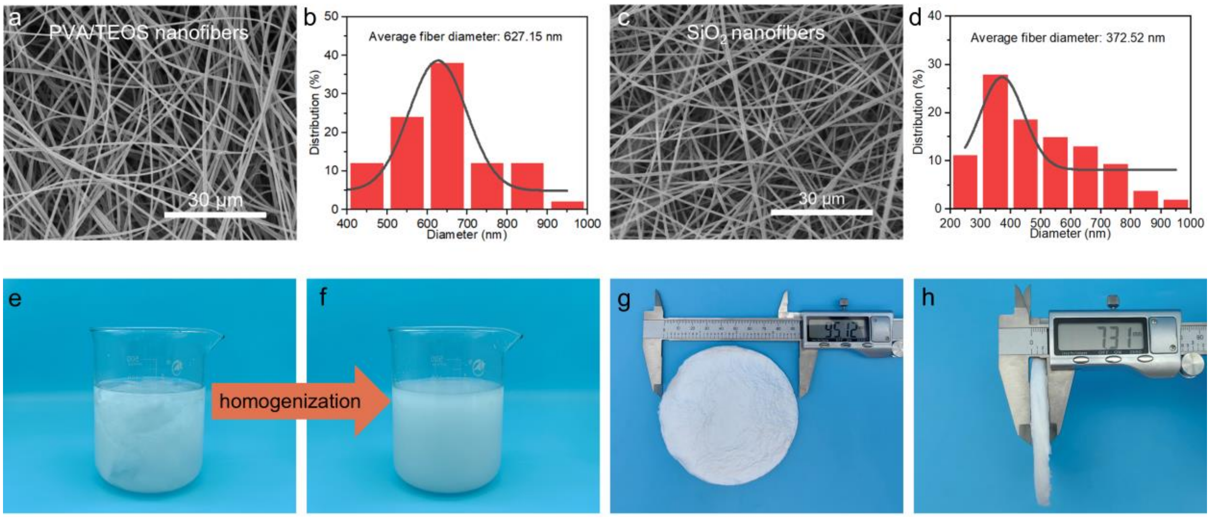

Figure S2. Morphology and photographs of the preparation process of homogenized SiO_2 nanofibers. (a) SEM image and (b) diameter distribution of electrospun PVA/TEOS nanofibers. (c) SEM image and (d) diameter distribution of electrospun SiO_2 nanofibers, Optical photographs showing the preparation process of the homogenized SiO_2 webs by high-speed homogenization: (e) SiO_2 nanofibrous membranes in water, (f) homogenized SiO_2 dispersions and, (g, h) homogenized SiO_2 nanofiber webs obtained by filtration.

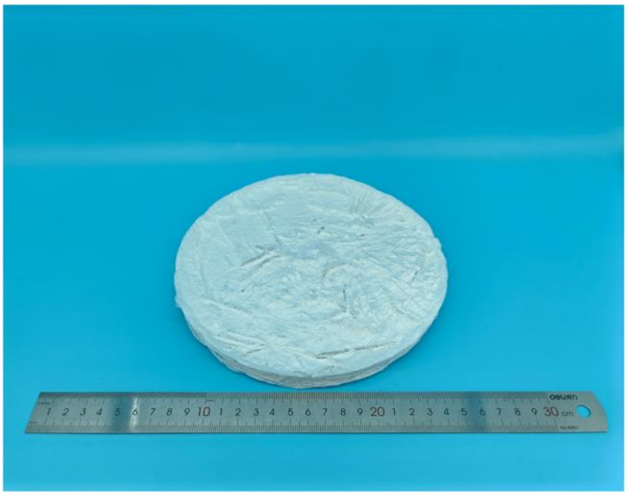

Figure S3. Photograph of a CNF/SNF aerogel (16 cm diameter).

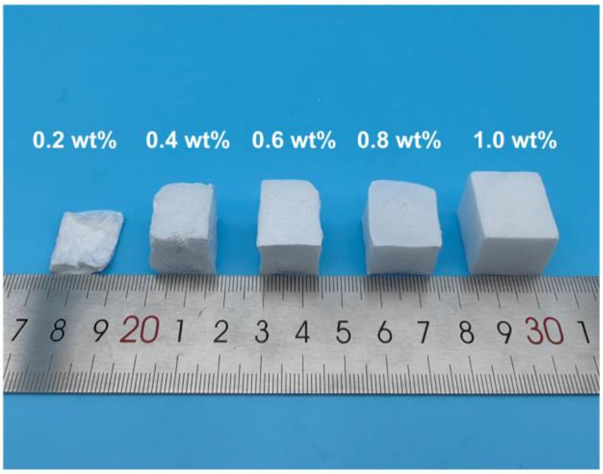

Figure S4. Photographs of CNF/SNF aerogels with given nanofiber content, ranging from 0.2 to 1.0 wt.%.

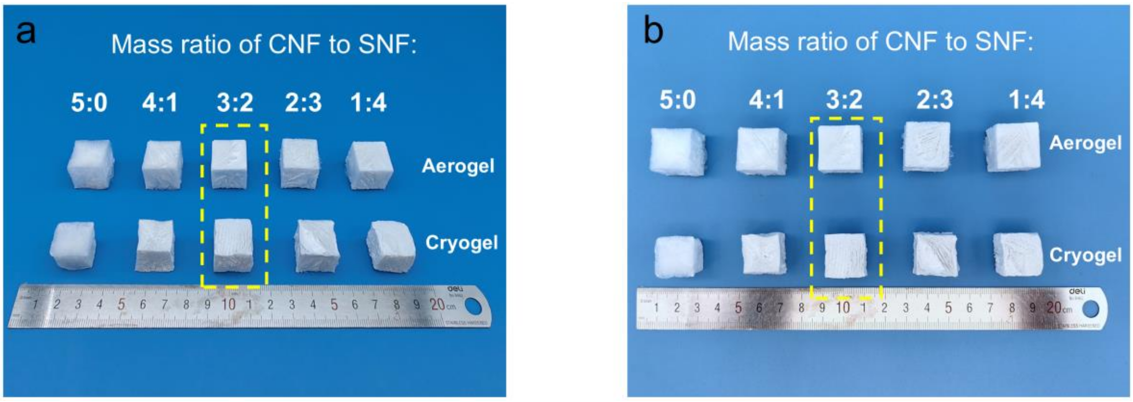

Figure S5. Aerogels and cryogels of different CNF:SNF ratios prepared by freeze-drying and ambient drying, respectively. At a fixed dispersion concentration of 1 wt.%, a CNF to SNF mass ratio of 3:2 was identified as optimal. This composition demonstrates volumetric shrinkage behavior comparable to freeze-drying, while maintaining outstanding structural integrity.

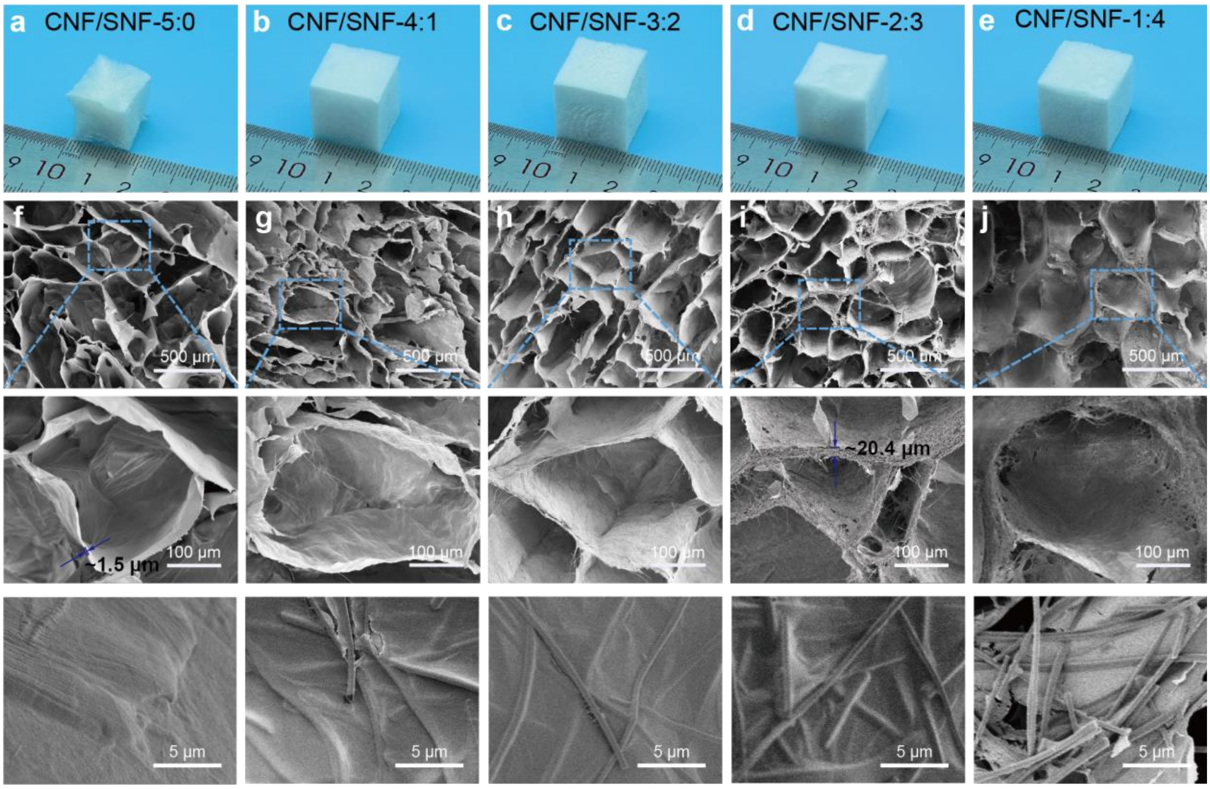

Figure S6. Morphology of CNF/SNF aerogels. Photographs and SEM images of aerogels (a, f) CNF/SNF-5:0, (b, g) CNF/SNF-4:1, (c, h) CNF/SNF-3:2, (d, i) CNF/SNF-2:3, (e, j) CNF/SNF-1:4. As the increase of the proportion of SNF, the pore size and wall thickness of the aerogel increase, which suggested that SNF has a regulatory effect on the aerogel structure

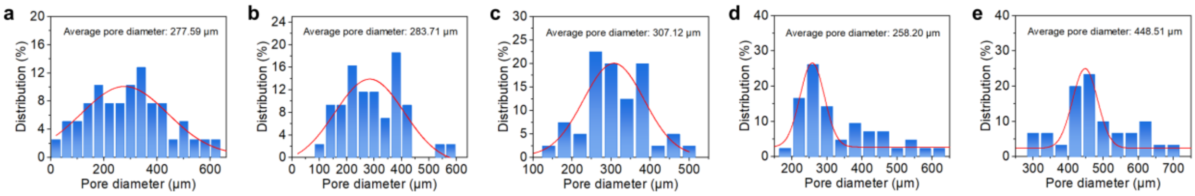

Figure S7. Pore diameter distributions of the aerogels: (a) CNF/SNF-5:0, (b) CNF/SNF-4:1, (c) CNF/SNF-3:2, (d) CNF/SNF-2:3 and (e) CNF/SNF-1:4.

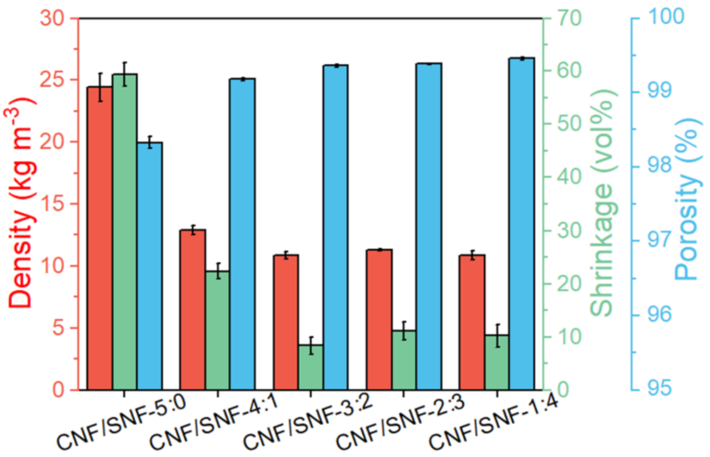

Figure S8. Density, volume shrinkage, and porosity of aerogels with different CNF and SNF ratios.

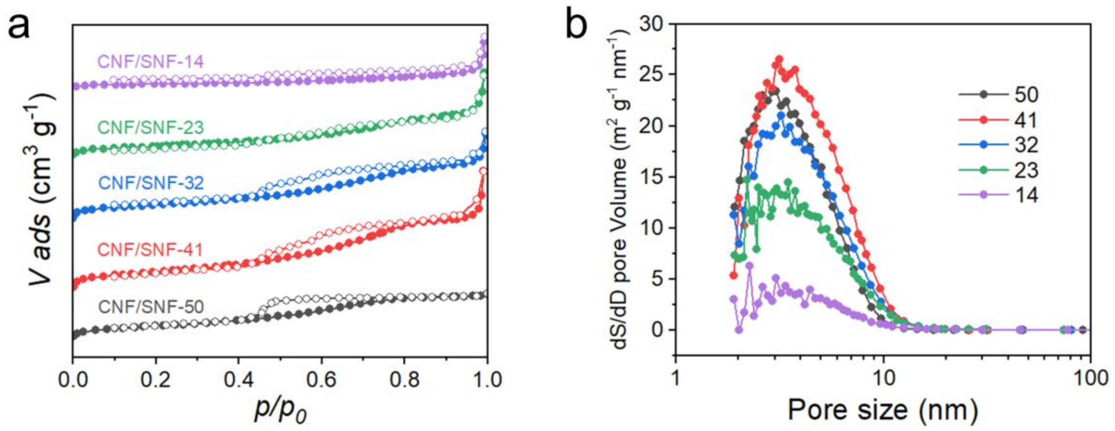

Figure S9. (a) Nitrogen adsorption isotherms and (b) pore-size distributions of CNF/SNF aerogels with various CNF and SNF ratios.

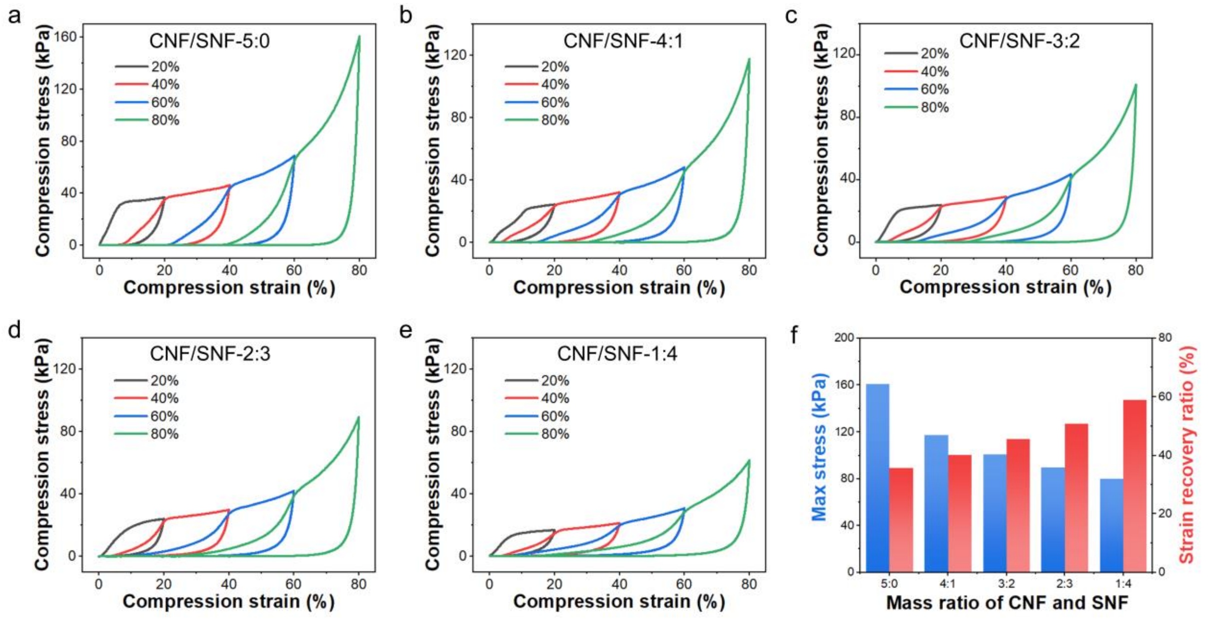

Figure S10. Stress–strain curves of CNF/SNF aerogels with various CNF:SNF ratios: (a) CNF/SNF-5:0, (b) CNF/SNF-4:1, (c) CNF/SNF-3:2, (d) CNF/SNF-2:3, and (e) CNF/SNF-1:4 at given maximum strains (%). (f) Maximum stress and strain recovery ratio of CNF/SNF aerogels.

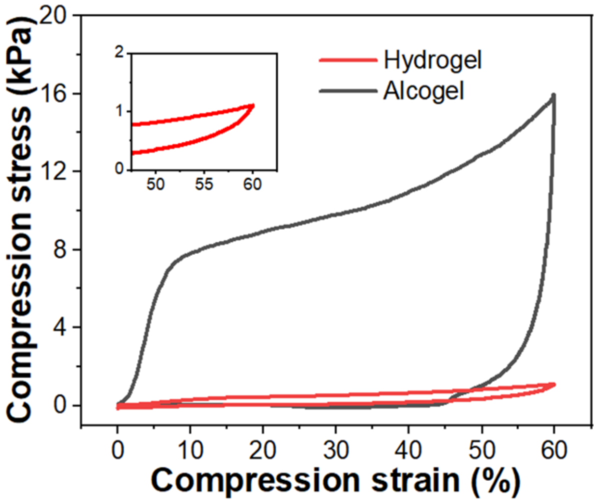

Figure S11. Stress–strain curves of CNF/SNF hydrogel and alcogel. Under the same 60% compressive strain, the maximum compressive strength of the CNF/SiO_2 alcogel is 16 kpa, approximately 14.5 times that of the CNF/SiO_2 hydrogel (1.1 kPa). Furthermore, the Young's modulus also increased significantly, from 3.2 kPa for hydrogel to 156.4 kPa for the alcogel.

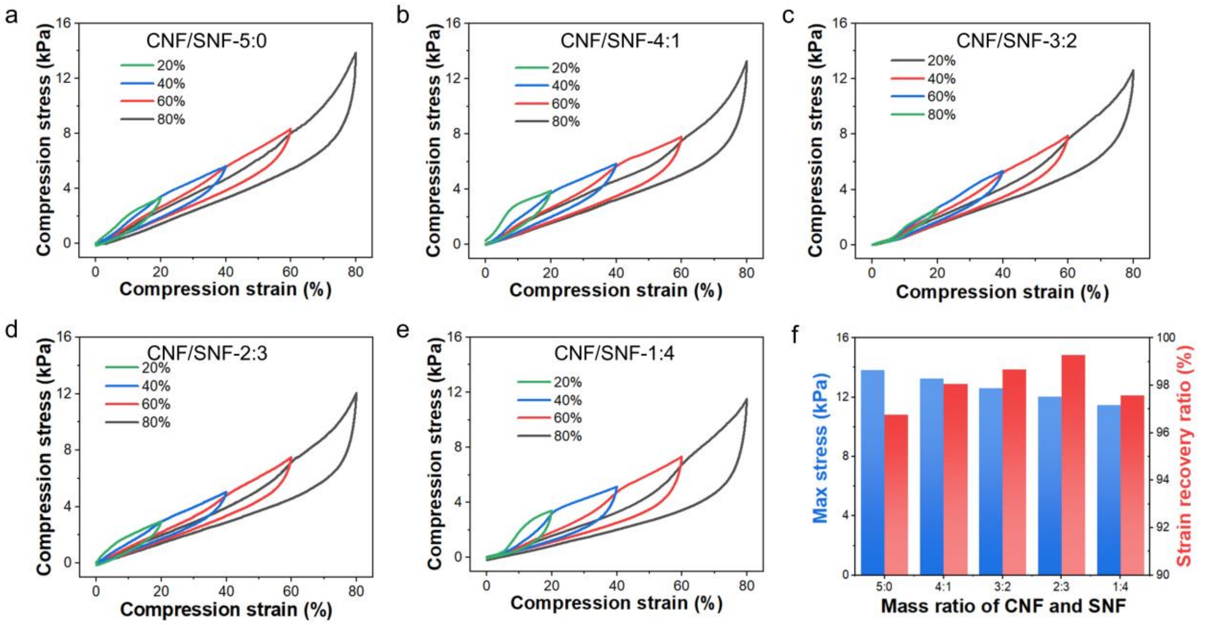

Figure S12. Underwater compression stress–strain curves of CNF/SNF aerogels with various CNF:SNF ratios: (a) CNF/SNF-5:0, (b) CNF/SNF-4:1, (c) CNF/SNF-3:2, (d) CNF/SNF-2:3, and (e) CNF/SNF-1:4 at given maximum strains. (f) Maximum stress and strain recovery of CNF/SNF aerogels.

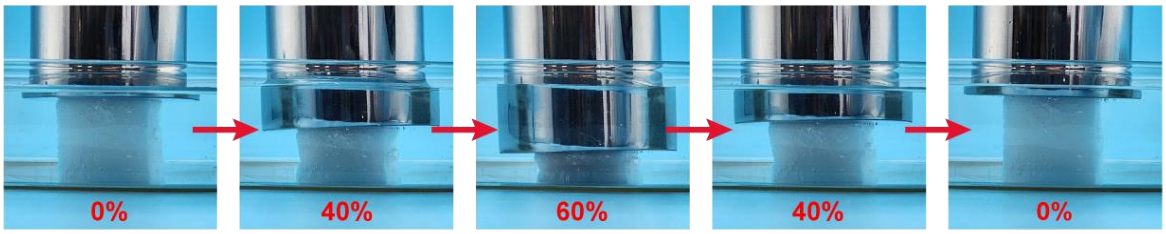

Figure S13. Photographs illustrating underwater compressive deformation and recovery of aerogels.

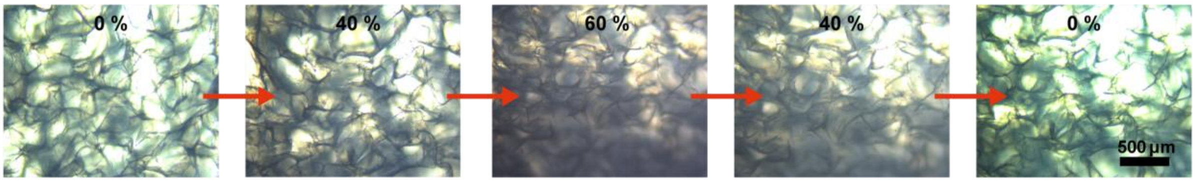

Figure S14. Optical images illustrating underwater compressive deformation and recovery of samples and aerogel's cell wall. During underwater compression, the cell walls gradually bend and fold, stacking upon one another and thereby reducing light transmittance. Upon release, water rapidly infiltrates the cellular network driven by capillary forces, and the deformed cell walls swiftly rebound to their original configuration.

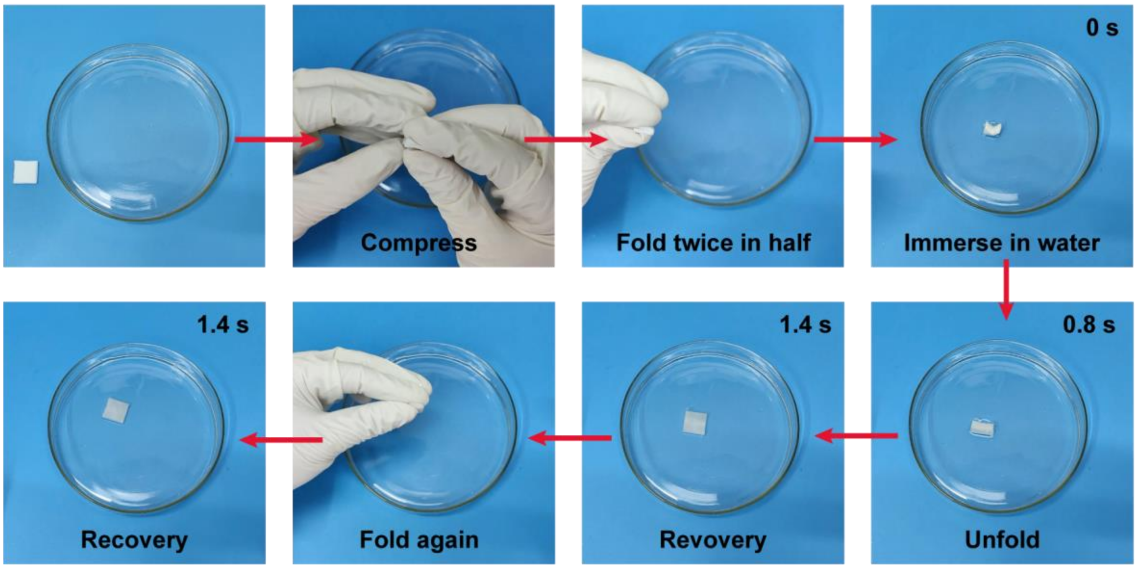

Figure S15. Photographs showing rapid shape recoverability of CNF/SNF aerogels after hydration. The aerogel was subjected to two cycles of folding and compression, after which it was immersed in water. It quickly absorbed water and expanded to its original dimensions. This reversible swelling behavior was retained even after multiple cycles of dehydration and mechanical compression, indicating outstanding shape recovery and structural integrity.

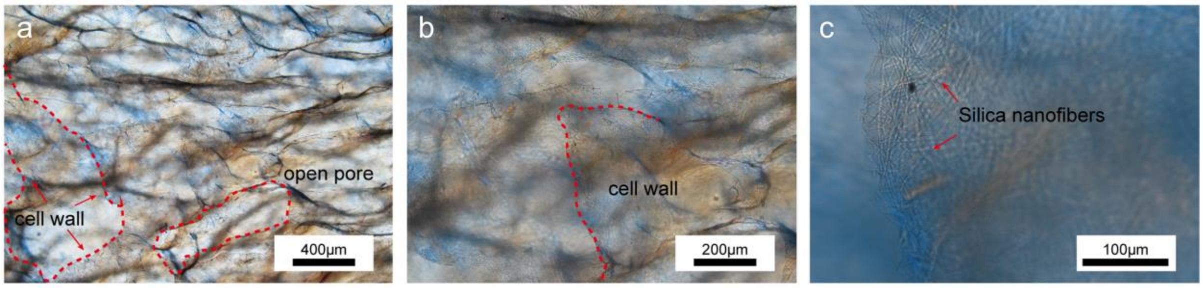

Figure S16. High-resolution photomicrographs of the nanofibrous cell wall reveal the open pore structure and cell wall of the aerogel. The silica nanofiber network embedded within the cell wall structure is distinctly visible under microscopic observation.

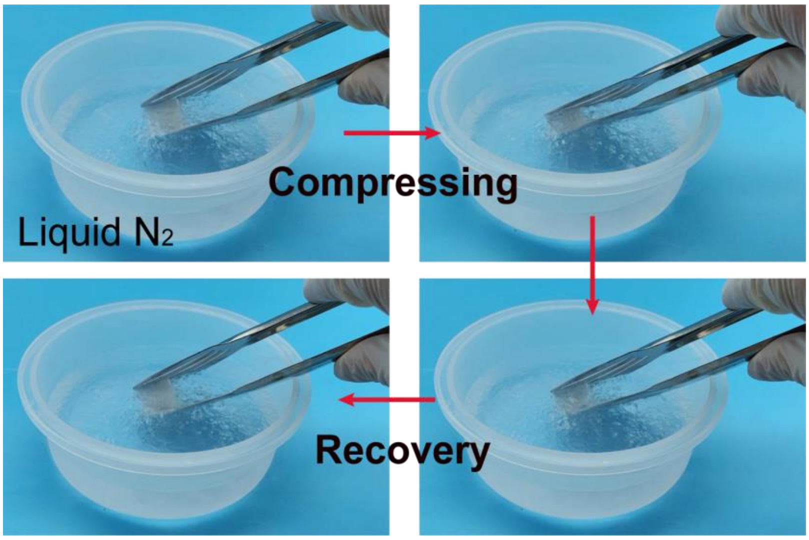

Figure S17. Photographs showing the elasticity of CNF/SNF aerogels immersed in liquid nitrogen. Aerogel still maintains compressible flexibility and elasticity at low temperatures.

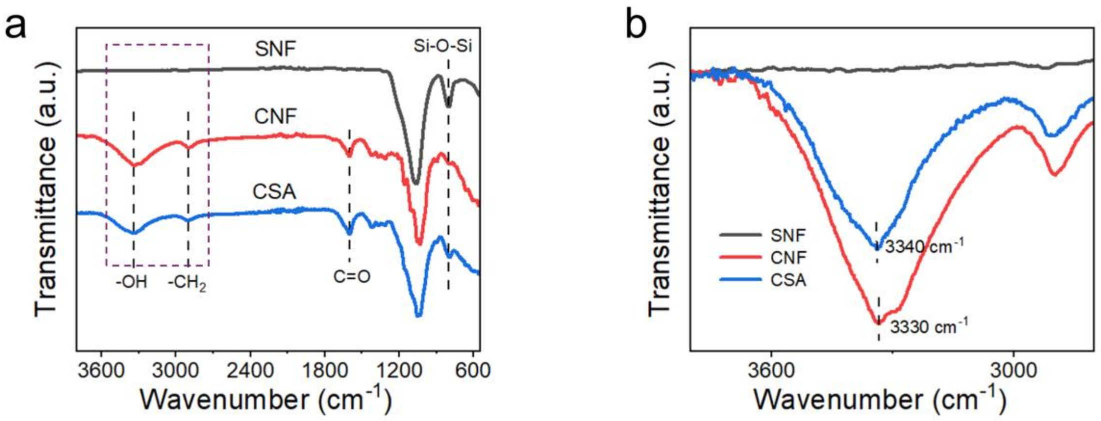

Figure S18. Fourier transform infrared (FTIR) spectra of SiO_2 nanofibers (SNF), CNF and CNF/SNF aerogels: (a) Full spectrum (3800-550 cm^{-1}) and (b) spectra in the 3800-2800 cm^{-1} region. The incorporation of SNF into the CNF network induced a slight shift in the hydroxyl peak, a typical indicator of a weakened hydrogen-bonding network.

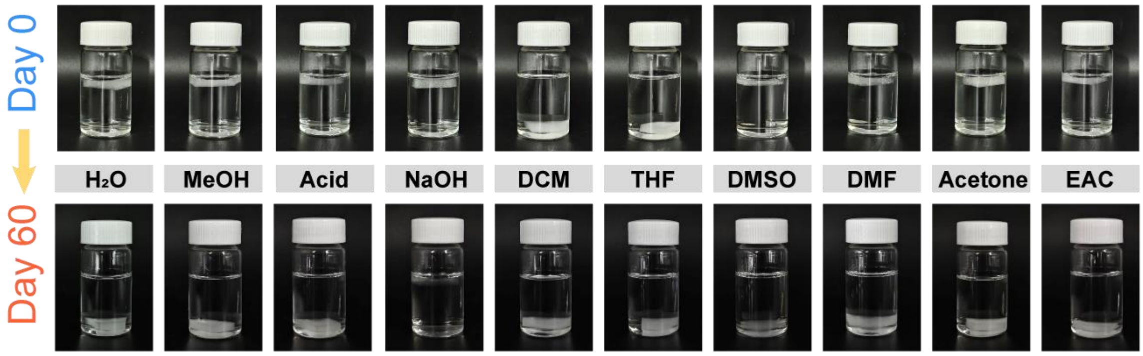

Figure S19. Wet and chemical stability of the of CNF/SNF aerogels in water, acidic and alkaline solutions, as well as common organic solvents tested for up to two months.

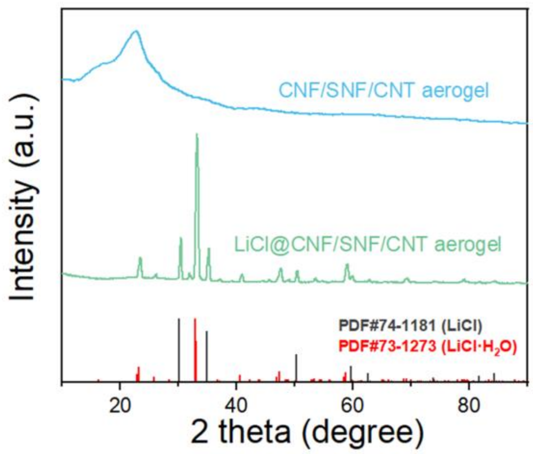

Figure S20. XRD patterns of CNF/SNF/CNT aerogel (bule curve) and LiCl@CNF/SNF/CNT aerogel (green curve).

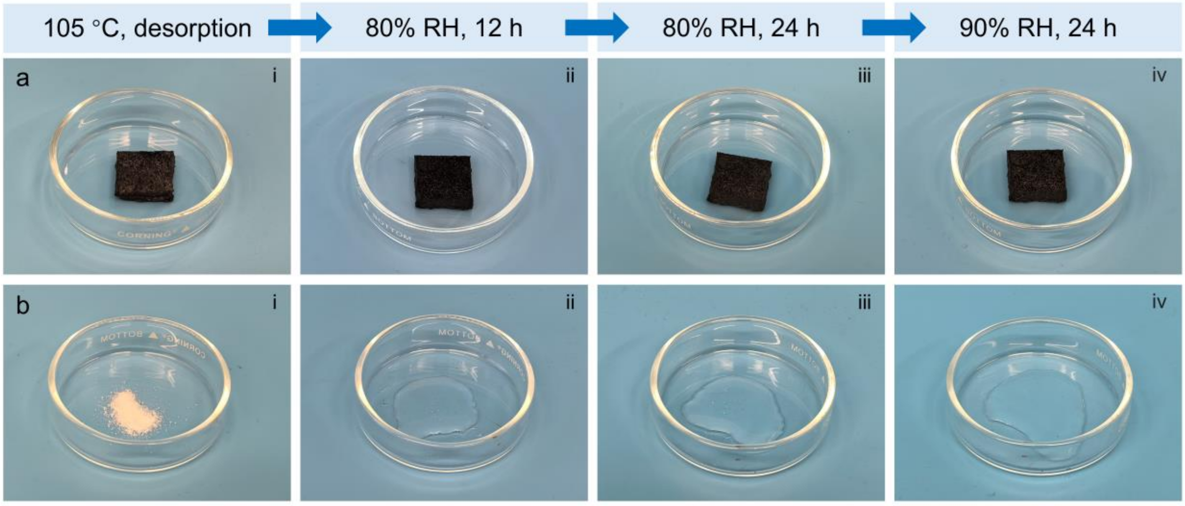

Figure S21. Photographs showing moisture absorption behavior of samples under high-humidity conditions. (a) LiCl@CCS aerogel and (b) neat LiCl particles (equal weight) compared for moisture absorption performance. The samples were subjected to the following conditions: (i) completely dried at 105 °C; (ii) exposed to 80% RH for 12 hours; (iii) exposed to 80% RH for 24 hours; and (iv) subsequently exposed to 90% RH for an additional 24 hours. While pure LiCl rapidly absorbs moisture and dissolves, the LiCl-loaded aerogel showed no visible leakage or overflow. This stability is attributed to the aerogel’s porous architecture, capillary retention, and superhydrophilic nature.

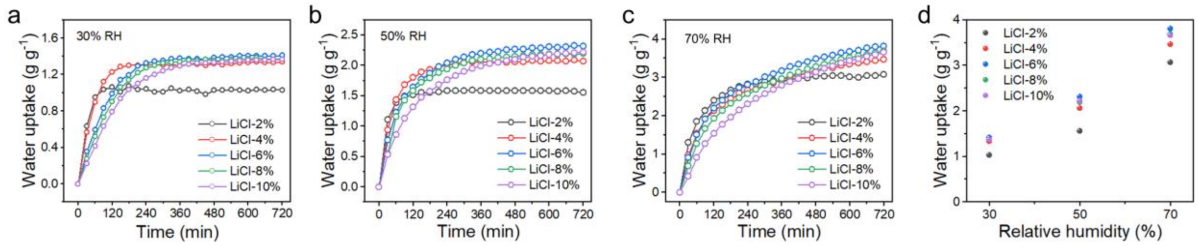

Figure S22. Water uptake of LiCl@CCS aerogels with various LiCl loadings under (a) 30%, (b) 50% and (c) 70% RH conditions within 12 h. (d) Water uptake of LiCl@CCS aerogels with various LiCl loadings under 30%, 50%, and 70% RH.

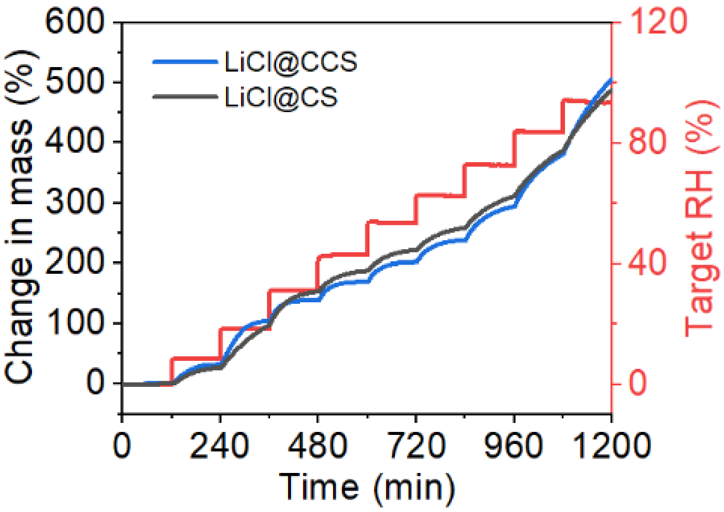

Figure S23. Stepwise moisture adsorption curves for LiCl@CCS and LiCl@CS at 25 °C. The results show that both aerogels exhibit nearly identical sorption profiles, with only minor differences.

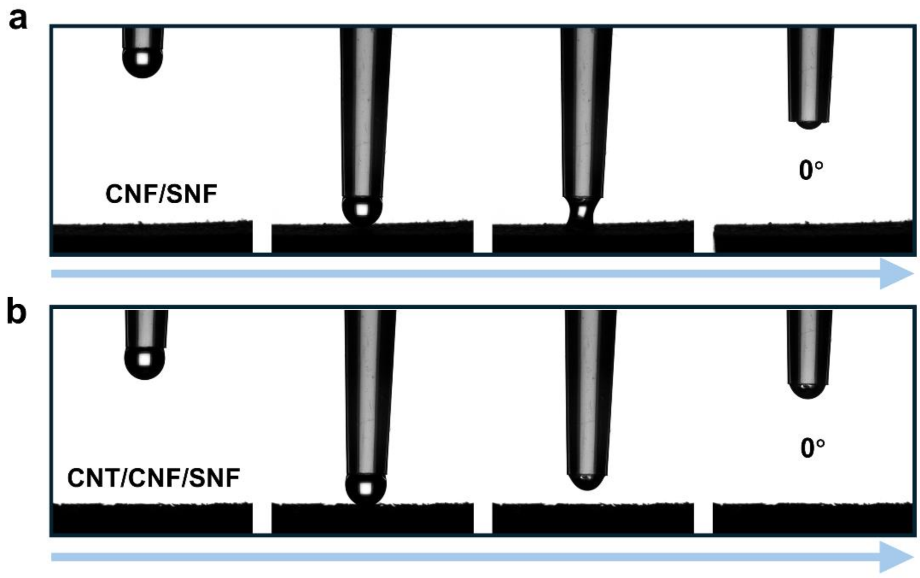

Figure S24. Superhydrophilicity of (a) CNF/SNF aerogel and (b) CNT/CNF/SNF aerogel. Both CNF/SNF and CNT/CNF/SNF aerogels exhibited superhydrophilic behavior, attributed to the strong water affinity of CNF and SNF. The small amount of CNT incorporated did not significantly affect the overall hydrophilicity. No visible changes were observed on the aerogel surface during the water absorption process.

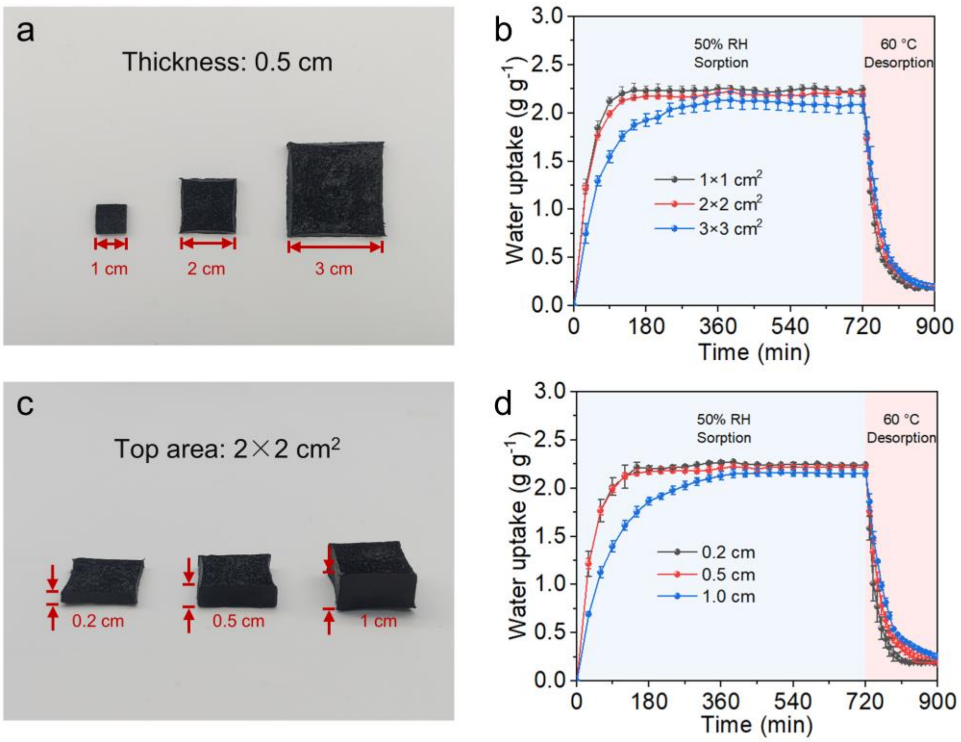

Figure 25. Effect of sample dimensions on moisture absorption and desorption behavior. (a) Photographs of aerogel samples with fixed thickness (0.5 cm) and varying areas (1×1, 2×2, 3×3 cm^2). (b) Dynamic adsorption–desorption curves of samples with different areas. (c) Photographs of aerogel samples with fixed area (2×2 cm^2) and varying thicknesses (0.2, 0.5, 1.0 cm). (d) Dynamic adsorption–desorption curves of samples with different thicknesses. Aerogels with surface areas of 1×1 cm^2 and 2×2 cm^2 showed comparable moisture absorption behavior, while the 3×3 cm^2 sample exhibited slower absorption, likely due to diffusion limitations within the larger structure. In terms of desorption, samples with the same thickness showed minimal differences, highlighting the dominant role of thickness in desorption kinetics. Regarding the effect of thickness, the 0.2 cm and 0.5 cm thick samples exhibited similar and significantly faster desorption rates compared to the 1.0 cm sample. Thinner samples consistently released moisture more rapidly. Based on these results, a thickness of 0.5 cm was found to offer an optimal balance between efficient moisture absorption/desorption and high-water uptake per unit mass.

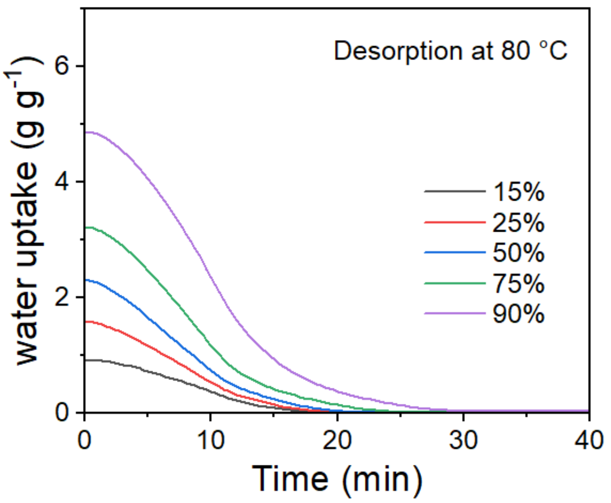

Figure S26. The desorption curves of the LiCl@CCS aerogels at 15% RH, 30% RH, 50% RH, 75% RH, and 90% RH. Under the desorption temperature condition of 80 °C, samples that absorb moisture at different humidity levels can all achieve rapid desorption within 30 min.

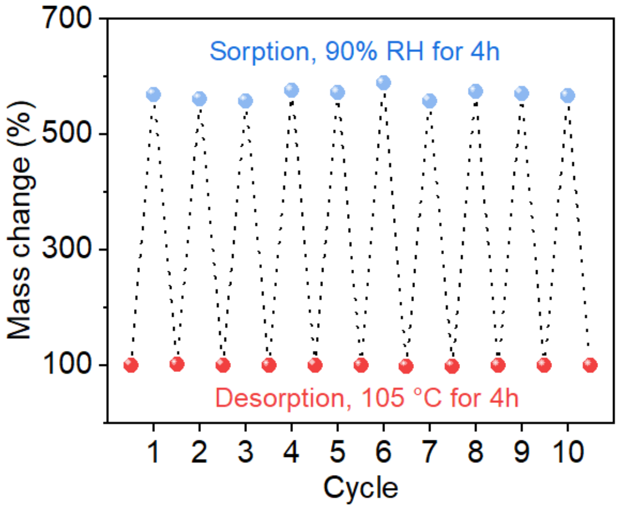

Figure S27. Sorption-desorption performance of LiCl@CCS aerogel over 10 rapid cycles under high humidity (90% RH) and accelerated desorption at 105 °C. The results demonstrate the aerogel's excellent stability and retention of sorption capacity under repeated cycling conditions.

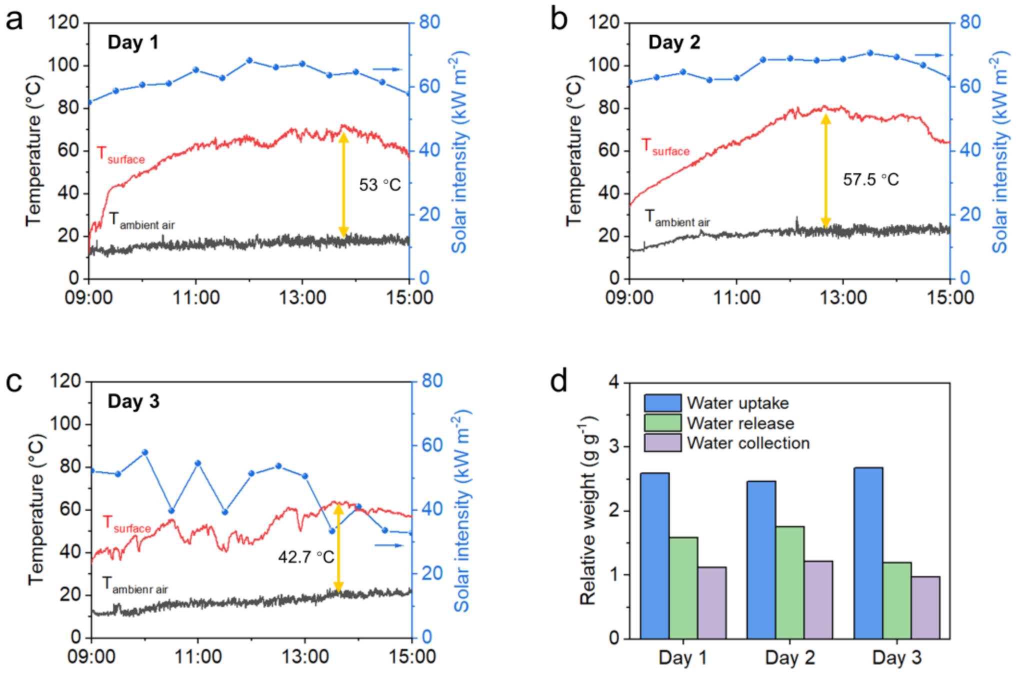

Figure S28. Solar-driven outdoor water harvesting tests conducted continuously over three days (April 8–10, 2025) in Harbin, China. (a–c) Solar intensity, ambient air temperature, and aerogel surface temperature recorded from 9:00 a.m. to 3:00 p.m. during Days 1–3. (d) Water uptake, water release, and total water collected over the three-day testing period. Despite fluctuations in humidity, temperature, and sunlight, the aerogels maintained stable water uptake and release, confirming their robust performance and practical applicability in real-world environments.

Supplementary Tables

Table S1. Density, volume shrinkage, porosity, and surface area of aerogels.

Samples	Density (mg cm^{-3})	Volume shrinkage (%)	Porosity (%)	Surface area (m^2/g)
CNF/SNF-5:0	24.41 ± 1.13	59.34 ± 2.21	98.33 ± 0.08	77.74
CNF/SNF-4:1	12.9 ± 0.36	22.36 ± 1.47	99.18 ± 0.02	104.21
CNF/SNF-3:2	10.86 ± 0.32	8.37 ± 1.62	99.36 ± 0.02	82.90
CNF/SNF-2:3	11.32 ± 0.06	11.14 ± 1.64	99.38 ± 0.03	58.78
CNF/SNF-1:4	10.89 ± 0.36	10.20 ± 2.15	99.45 ± 0.02	20.45

Table S2. Summary of biomass-based ambient pressure drying aerogels.

**Skeleton material**	**Crosslinker**	**Drying solvent**	**Minimum density, mg cm^{-3}**	**Ref.**
CNFs/Mxene	PDMI	Acetone	18	[1]
alginate/PEDOT:PSS	PANI	EtOH	38.5	[2]
CNF	TA-Metal ions	EtOH	~18	[3]
Cellulose/BT	BDE	H_2O	90	[4]
CNF/CMC	DMTMM	Acetone	29.4	[5]
Chitosan	ECH	EtOH	–	[6]
CMCNa	Cu^{2+}	EtOH	>20	[7]
CNF/Alginate	Ca^{2+}	Acetone	24	[8]
CNF	b-PEI/GPTMS	Acetone	58.82	[9]
MCNFs/TOCNFs	Fe^{3+}	EtOH	–	[10]
Cellulose/Graphite	Metal ions	SDS/H_2O	36	[11]
CNF/Flake graphite	Ca^{2+}	SDS/H_2O	36	[12]
CNF/Alginate	Ca^{2+}	Acetone	21.6	[13]
**CNF/SNF**	**–**	**EtOH**	**10.86**	**This work**

Key: PMDI: poly ((phenyl isocyanate)-*co*-formaldehyde); PANI: polyaniline; TA: tannic acid; BT: bentonite; BDE: 1,4-Butanediol diglycidyl ether; CMCs: carboxymethyl chitosan; DMTMM: a triazine derivative, 4-(4,6-Dimethoxy[1.3.5]triazin-2-yl)-4-methylmorpholinium chloride hydrate; ECH: Epichlorohydrin; CMCNa: sodium carboxymethyl cellulose; b-PEI: Branched polyethylenimine; GPTMS: g-Glycidoxypropyltrimethoxysilane; MCNFs: mechanical-isolated cellulose nanofibrils.

References

(1) Wu, N.; Yang, Y.; Wang, C.; Wu, Q.; Pan, F.; Zhang, R.; Liu, J.; Zeng, Z. Ultrathin Cellulose Nanofiber Assisted Ambient-Pressure-Dried, Ultralight, Mechanically Robust, Multifunctional MXene Aerogels. *Adv. Mater.* **2023**, *35* (1), 2207969.

(2) Zhang, Y.-C.; Ding, R.; Su, P.-G.; Zeng, F.-R.; Jia, X.-X.; Hu, Z.-Y.; Wang, Y.-Z.; Zhao, H.-B. Biomimetic Ambient-Pressure-Dried Aerogels with Oriented Microstructures for Enhanced Electromagnetic Shielding. *Adv. Funct. Mater.* **2025**, *35* (5), 2414683.

(3) Mattos, B. D.; Zhu, Y.; Tardy, B. L.; Beaumont, M.; Ribeiro, A. C. R.; Missio, A. L.; Otoni, C. G.; Rojas, O. J. Versatile Assembly of Metal–Phenolic Network Foams Enabled by Tannin–Cellulose Nanofibers. *Adv. Mater.* **2023**, *35* (12), 2209685.

(4) Chen, L.; Wang, S.; Wang, S.; Chen, C.; Qi, L.; Yu, L.; Lu, Z.; Huang, J.; Chen, J.; Wang, Z.; et al. Scalable Production of Biodegradable, Recyclable, Sustainable Cellulose–Mineral Foams via Coordination Interaction Assisted Ambient Drying. *ACS Nano* **2022**, *16* (10), 16414-16425.

(5) Tang, S.; Ma, M.; Zhang, X.; Zhao, X.; Fan, J.; Zhu, P.; Shi, K.; Zhou, J. Covalent Cross-Links Enable the Formation of Ambient-Dried Biomass Aerogels through the Activation of a Triazine Derivative for Energy Storage and Generation. *Adv. Funct. Mater.* **2022**, *32* (36), 2205417.

(6) Qi, L.; Mu, L.; Guo, X.; Liu, A.; Chen, C.; Ye, Q.; Zhong, Z.; Shi, X. Fast Expandable Chitosan-Fibers Cryogel from Ambient Drying for Noncompressible Bleeding Control and In Situ Tissue Regeneration. *Adv. Funct. Mater.* **2023**, *33* (16), 2212231.

(7) Ni, Y.; Zhou, X.; Gong, J.; Xue, L.; Zhao, Q. Lyophilization-Free Engineering of Polyelectrolyte Monolith by an Ice-Dissolving-Complexation Method. *Adv. Funct. Mater.* **2021**, *31* (35), 2103818.

(8) Françon, H.; Wang, Z.; Marais, A.; Mystek, K.; Piper, A.; Granberg, H.; Malti, A.; Gatenholm, P.; Larsson, P. A.; Wågberg, L. Ambient-Dried, 3D-Printable and

Electrically Conducting Cellulose Nanofiber Aerogels by Inclusion of Functional Polymers. *Adv. Funct. Mater.* **2020**, *30* (12), 1909383.

(9) Li, Y.; Grishkewich, N.; Liu, L.; Wang, C.; Tam, K. C.; Liu, S.; Mao, Z.; Sui, X. Construction of functional cellulose aerogels via atmospheric drying chemically cross-linked and solvent exchanged cellulose nanofibrils. *Chem. Eng. J.* **2019**, *366*, 531-538.

(10) Li, J.; Chen, S.; Li, X.; Zhang, J.; Nawaz, H.; Xu, Y.; Kong, F.; Xu, F. Anisotropic cellulose nanofibril aerogels fabricated by directional stabilization and ambient drying for efficient solar evaporation. *Chem. Eng. J.* **2023**, *453*, 139844.

(11) Wang, R.; Chen, C.; Pang, Z.; Wang, X.; Zhou, Y.; Dong, Q.; Guo, M.; Gao, J.; Ray, U.; Xia, Q.; et al. Fabrication of Cellulose–Graphite Foam via Ion Cross-linking and Ambient-Drying. *Nano Lett.* **2022**, *22* (10), 3931-3938.

(12) Zong, S.; Feng, C.; Lei, F.; Zhu, L.; Jiang, J.; Duan, J. Construction of Nanocellulose Aerogels with Environmental Drying Strategy without Organic Solvent Displacement for High-Efficiency Solar Steam Generation. *ACS Nano* **2025**, *19* (5), 5305-5315.

(13) Rostami, J.; Gordeyeva, K.; Benselfelt, T.; Lahchaichi, E.; Hall, S. A.; Riazanova, A. V.; Larsson, P. A.; Cinar Ciftci, G.; Wågberg, L. Hierarchical build-up of bio-based nanofibrous materials with tunable metal–organic framework biofunctionality. *Mater. Today* **2021**, *48*, 47-58.
